# Silver (I) *N*-Heterocyclic Carbenes Carbosilane Dendritic Systems and Their Imidazolium-Terminated Analogues as Antibacterial Agents: Study of Their Mode of Action

**DOI:** 10.3390/pharmaceutics12100968

**Published:** 2020-10-14

**Authors:** Tamara Rodríguez-Prieto, Philipp F. Popp, José Luis Copa-Patiño, F. Javier de la Mata, Jesús Cano, Thorsten Mascher, Rafael Gómez

**Affiliations:** 1 Department of Organic and Inorganic Chemistry, Chemical Research Institute “Andrés M. Del Río” (IQAR), University of Alcalá, 28805 Madrid, Spain; tamara.rodriguezp@edu.uah.es (T.R.-P.); javier.delamata@uah.es (F.J.d.l.M.); jesus.cano@uah.es (J.C.); 2 Ramón y Cajal Health Research Institute (IRYCIS), 28034 Madrid, Spain; 3 Networking Research Center on Bioengineering, Biomaterials and Nanomedicine (CIBER-BBN), 28029 Madrid, Spain; 4 Institute of Microbiology, Dresden University of Technology, 01069 Dresden, Germany; philipp.popp@tu-dresden.de; 5 Department of Biomedicine and Biotechnology, University of Alcalá, 28805 Madrid, Spain; josel.copa@uah.es

**Keywords:** dendrimers, carbosilane, carbene, silver, imidazolium, antibacterial activity, cell membrane depolarization, whole-cell biosensor

## Abstract

Spherical dendrimers and dendrons containing silver(I) *N*-heterocyclic carbenes (Ag(I)-NHC) and additionally bow-tie metal-free dendritic systems were synthesized in a simple and straightforward synthetic procedure and subsequently characterized. The antibacterial activity was evaluated, and in parallel, a comparative study with the cationic analogue precursors was performed to explore the effect of silver ions in the dendritic structure. Other parameters, such as topology, generation, and hydrophobicity, of the imidazole substituents were also studied. All these dendritic systems presented antibacterial activity against three different bacterial strains, two Gram-positive (*Staphylococcus aureus* and *Bacillus subtilis*) and one Gram-negative (*Escherichia coli*). Several assays were conducted to elucidate their mechanism of action against *Bacillus subtilis*, by using bacterial biosensors or specific probes and fluorescent proteins sensitive to changes in the cell membrane potential. These studies are specially focused on the role of the polyvalence of our systems containing silver atoms, which may provoke interesting effects in the mode of action.

## 1. Introduction

One of the greatest threats to society is the incidence of invasive infectious microbial diseases, causing a worldwide problem. Besides that, the possible development of antibiotic resistance could generate a more critical situation. This is the reason why there is a special need in developing new specific antimicrobial systems with different modes of action, which help to eradicate these infections. Recently, antimicrobial peptides [1,2] and cationic polymers [3,4] have received great attention because of their broad spectrum of action against different bacterial strains. These antimicrobial agents normally contain cationic moieties as well as hydrophobic side chains. Among the wide range of new molecules involved in drug discovery, the presence of imidazolium salts has aroused a special interest, due to their high therapeutic properties [5,6]. They are used in different medical areas, such as, anticancer, anti-inflammatory, antibacterial, antifungal, or antiviral [7,8,9,10], demonstrating promising results. Their activity is provided by the azole core, the ionic structure, and the substituents [6]. Due to the versatility of the heterocyclic structure it provides, it is possible to attach to the five-membered planar ring a wide range of molecular fragments, leading to combined different properties in one single molecule. Moreover, imidazole is a very versatile group widely used as precursors of N-heterocyclic carbenes (NHCs) [11]. Usually, NHCs are obtained through an in situ deprotonation of imidazolium salts, capable of leading to a strong bonding in several transition metals [12]. Commonly, NHC ligands act as a high σ-donor and low π-acceptor, providing stability to the metal center [13]. Ag(I)-NHC are widely used in transmetalation chemistry; however, their successful applications in biological studies have resulted in focused efforts in this direction [14].

Since ancient times, the microbiological properties of silver ions have been generally known, and they are present in many microbicide products. While the mechanism of action is not completely clarified [15], an advantage of using silver as a microbicide is that no resistances are found.

Currently, another interesting property considered in developing new microbicides agents is the polyvalency of the molecule, i.e., having the possibility to attach several therapeutic agents in their structure and therefore potentially providing higher microbicide activity [16]. Polymers are extensively used as microbicides because of this unique feature [17]. Among them, dendrimers conceived as non-conventional polymers or macromolecules are of particular interest as they can be applied as a multivalent platform [18]. In contrast to conventional polymers, their main advantages are that they are ideally monodisperse and their synthesis can be controlled, enabling design of the final structure and selective attachment of different functional groups in a precise manner [19,20]. In this way, it is possible to make use of a wide range of dendrimers, with different typologies, topologies (shapes), and terminal functional groups, and give them a specific function [19,21,22]. In this sense, just a few works have been reported in the use of dendrimers containing silver atoms [23,24].

Carbosilane dendrimers are one type of dendritic structures based on silicon-carbon bonds, providing thermal and chemical stability. These properties as well as the biocompatibility showed for these systems are really advantageous for medical application [25,26].

This work focused on the synthesis of a new family of cationic carbosilane dendritic systems containing imidazolium salts and their analogues with Ag(I)-NHC fragments in the periphery. For all these synthesized systems, their antibacterial properties against *Escherichia coli*, *Staphylococcus aureus*, and *Bacillus subtilis* were studied in detail by determining the minimal inhibitory concentration (*MIC*) and the minimal bactericidal concentration (*MBC*). Subsequently, we focused on unraveling the detailed mode of action of the synthesized compounds against the Gram-positive model organism *B. subtilis*. First, we tested a panel of whole-cell biosensors to narrow down the cellular target [27]. After we identified the cell envelope as the main target of the tested compounds, we comprehensively analyzed the mechanism and uncovered their detailed cell membrane-perturbating activity.

## 2. Experimental Section

All reactions were performed using inert conditions and dry solvents when necessary. Ag_2_O was obtained from commercial sources (Alfa Aesar, Karlsruhe, Germany, 99+%) and imidazolium-terminated dendrimers **I**–**V**, amine-terminated dendrons with imidazolium groups at the focal point **VI**–**VII** [28], and allyl-terminated bow-tie dendrimers **VIII**–**IX**, previously synthesized in our research group, were prepared as described in the literature [21]. Two types of N-substituents in the NHC ligand were studied, methyl and mesityl groups, and the abbreviation MeImid and MesImid used respectively to refer to them. All reactions were followed by Varian Unity VXR-300 NMR spectra (Agilent Technologies, Palo Alto, CA, USA) at ambient temperature, through ^1^H and ^13^C resonances and Heteronuclear Single-Quantum Correlation (HSQC), Heteronuclear Multiple Bond Correlation (HMBC), Bidimensional proton/proton Correlation Spectroscopy (COSY), Totally Correlated Spectroscopy (TOCSY) NMR experiments, when necessary. Elemental analyses were performed on a PerkinElmer 240C instrument (PerkinElmer, Walham, MS, USA). Mass spectra (MS) were obtained from an Agilent 6210 spectrometer (ESI, Agilent Technologies, Palo Alto, CA, USA) in the positive mode. Attempts to carry out MS to silver (I) containing dendrimers and dendron failed. To obtain optical density (*OD*_600_), Ultrospec 2100 pro (Thermo Fisher, Waltham, MA, USA) and 1 cm^2^ cuvettes were used.

### 2.1. Synthesis of Ag-NHC-Terminated Spherical Dendrimers

#### 2.1.1. Synthesis of G_1_Si(CH_2_MeImidAgCl)_4_ (**1**)

Ag_2_O (0.024 g, 0.1 mmol) was added to a solution of G_1_Si(CH_2_MeImidCl)_4_ (**I**) (0.05 g, 0.052 mmol) in dry dichloromethane. It was stirred overnight at room temperature and protected from the light. After filtration and removal of the solvents, a dark oil was obtained (66.05 mg, 91%). ^1^H-NMR (CDCl_3_): δ(ppm) 6.99, 6.91 (m, 8H, NC*H*C*H*N), 3.79 (s, 12H, NC*H_3_*), 3.76 (s, 8H, SiC*H_2_*N), 1.27 (m, 8H, CH_2_C*H_2_*CH_2_), 0.68 (m, 8H, CH_3_SiC*H*_2_), 0.53 (m, 8H, SiC*H*_2_CH_2_), 0.08 (s, 24H, SiC*H*_3_). ^13^C[3]-NMR (CDCl_3_): δ (ppm) 178.9 (N*C*N), 121.8 (N*C*H*C*HN), 43.2 (Si*C*H*_2_*N), 38.6 (N*C*H*_3_*), 18.8–17.2 (Si*C*H_2_*C*H*_2_*), −3.8 (Si*C*H_3_). Elemental analysis calculated (%) for C_40_H_80_Ag_4_Cl_4_N_8_Si_5_ (1386.86): C 34.64, H 5.84, N 8.08; found C 35.09, H 5.73, N 8.15.

#### 2.1.2. Synthesis of G_2_Si(CH_2_MeImidAgCl)_8_ (**2**)

The synthetic method to obtain this compound was the same as previously described for **1**, using G_2_Si(CH_2_MeImidCl)_8_ (**II**) (0.42 g, 0.02 mmol) and Ag_2_O (17.5 mg, 0.08 mmol). Compound **2** was obtained as dark powder (49.6 mg, 80%). ^1^H-NMR (CDCl_3_): δ (ppm) 7.01, 6.92 (m, 16H, NC*H*C*H*N), 3.78 (s, 24H, NC*H_3_*), 3.74 (s, 16H, SiC*H_2_*N), 1.27 (m, 8H, CH_2_C*H_2_*CH_2_), 0.66, 0.52 (m, 48H, SiC*H*_2_), 0.07 (s, 48H, Si(*C*H_3_)_2_), −0.11 (s,12H, Si*C*H_3_). ^13^C[3]-NMR (CDCl_3_): δ (ppm) 178.6 (N*C*N), 122.6, 122.1 (N*C*H*C*HN), 43.5 (Si*C*H*_2_*N), 38.9 (N*C*H*_3_*), 19.3–17.9 (Si*C*H_2_*C*H*_2_*), −3.4, −4.7 (Si*C*H_3_). Elemental analysis calculated (%) for C_96_H_196_Ag_8_Cl_8_N_16_Si_13_ (3086.39): C 37.36, H 6.40, N 7.26; found C 37.27, H 6.24, N 7.93.

#### 2.1.3. Synthesis of G_3_Si(CH_2_MeImidAgCl)_16_ (**3**)

To obtain compound **3**, the same procedure as that for **1** was used, adding G_3_Si(CH_2_MeImidCl)_16_ (**III**) (0.027 g, 0.006 mmol) and Ag_2_O (0.011 g, 0.045 mmol). Compound **3** was obtained as a black powder (28.5 mg, 77%). ^1^H-NMR (CDCl_3_): δ (ppm) 7.05, 6.94 (m, 32H, NC*H*C*H*N), 3.79, 3.74 (s, 80H, NC*H_3_* and SiC*H_2_*N), 1.25 (m, 56H, CH_2_C*H_2_*CH_2_), 0.66, 0.56 (m, 64H, SiC*H*_2_), 0.07 (s, 96H, Si(*C*H_3_)_2_), -0.11 (s, 36H, Si*C*H_3_). ^13^C [3]-NMR (CDCl_3_): δ (ppm) 179.2 (N*C*N), 122.2, 121.9 (N*C*H*C*HN), 43.2 (Si*C*H*_2_*N), 38.7 (N*C*H*_3_*), 19.3–18.2 (Si*C*H_2_*C*H*_2_*), −3.7, −3.8, −4.9 (Si*C*H_3_). Elemental analysis calculated (%) for C_208_H_428_Ag_16_Cl_16_N_32_Si_29_ (6485.49): C 38.52, H 6.65, N 6.91; found C 38.24, H 6.52, N 8.09.

#### 2.1.4. Synthesis of G_1_Si(CH_2_MesImidAgCl)_4_ (**4**)

The synthetic method to obtain this compound was the same as that for **1**, using G_1_Si(CH_2_MesImidCl)_4_ (**IV**) (30.4 mg, 0.02 mmol) and Ag_2_O (10.3 mg, 0.04 mmol). Compound **4** was obtained as black powder (33.7 mg, 84%). ^1^H-NMR (CDCl_3_): δ (ppm) 7.25, 7.12 (m, 8H, NC*H*C*H*N), 6.92 (s, 8H, C*H_arom_*), 3.89 (s, 8H, SiC*H_2_*N), 2.32 (s, 12H, C*H_3arom_*), 1.96 (s, 24H, C*H_3arom_*), 1.38 (m, 8H, CH_2_C*H_2_*CH_2_), 0.76, 0.62 (m, 16H, SiC*H*_2_), 0.14 (s, 24H, SiC*H*_3_). ^13^C[3]-NMR (CDCl_3_): δ (ppm) 178.0 and 181.1 (N*C*N, coupling with ^109^Ag and ^107^Ag), 149.0–129.3 (*C*H*_arom._* and *C_ipso_*), 122.5 (NC*H*C*H*N), 43.4 (Si*C*H*_2_*N), 21.2–17.4 (*C*H*_3arom._* and Si*C*H_2_*C*H*_2_*), −3.6 (Si(*C*H_3_)_2_). Elemental analysis calculated (%) for C_72_H_112_Ag_4_Cl_4_N_8_Si_5_ (1803.44): C 47.95, H 6.26, N 6.21; found C 48.24, H 6.66, N 7.46.

#### 2.1.5. Synthesis of G_2_Si(CH_2_MesImidAgCl)_8_ (**5**)

The synthetic procedure was the same of that used to synthesize **1**, using G_2_Si(CH_2_MesImidCl)_8_ (**V**) (15 mg, 0.005 mmol) and Ag_2_O (4.5 mg, 0.02 mmol). The final product **5** was obtained as a dark powder (15.6 mg, 82%). ^1^H-NMR (CDCl_3_): δ (ppm) 7.24, 7.16 (m, 16H, NC*H*C*H*N), 6.91 (s, 16H, C*H_arom_*), 3.93 (s, 16H, SiC*H_2_*N), 2.30 (s, 24H, CH_3*arom*_), 1.94 (s, 42H, CH_3*arom*_.), 1.37 (m, 24H, CH_2_C*H_2_*CH_2_), 0.72, 0.58 (m, 48H, SiC*H*_2_), 0.12 (s, 48H, Si(C*H*_3_)_2_), 0.05 (s, 12H, SiC*H*_3_). ^13^C[3]-NMR (CDCl_3_): δ (ppm) 178.1 and 180.8 (N*C*N, coupling with ^109^Ag and ^107^Ag), 139.2–129.4 (*C*H*_arom_*. and *C_ipso_*), 122.7, 122.1 (NC*H*C*H*N), 43.2 (Si*C*H*_2_*N), 21.0–17.6 (*C*H_3*arom*_., Si*C*H_2_*C*H_2_), −3.8 (Si(*C*H_3_)_2_), −4.9 (Si*C*H_3_). Elemental analysis calculated (%) for C_160_H_260_Ag_8_Cl_8_N_16_Si_13_ (3919.6): C 49.75, H 6.66, N 5.70; found C 49.03, H 6.69, N 5.72.

### 2.2. Synthesis of Amine-Terminated Dendrons with Ag-NHC at the Focal Point

#### 2.2.1. Synthesis of AgClMeImidG_2_(S(CH_2_)_2_NMe_2_)_4_ (**6**)

To prepare compound **6**, the same method as that for **1** was used, using compound MeImidG_2_(S(CH_2_)_2_NMe_2_)_4_ (**VI**) (20 mg, 0.022 mmol) and Ag_2_O (6 mg, 0.022 mmol). Compound AgClMeImidG_2_(S(CH_2_)_2_NMe_2_)_4_ (**6**) was obtained as brown oil (24 mg, 89%). ^1^H-NMR (CDCl_3_): δ(ppm) 6.97, 6.95 (m, 2H, NC*H*C*H*N), 4.06 (t, 2H, J = 7.7 Hz, NC*H*_2_), 3.80 (s, 3H, NC*H*_3_), 2.55 (m, 24H, *CH_2_*S*CH_2_CH_2_*N), 2.24 (s, 24H, N(*CH_3_*)_2_), 1.75 (m, 2H, NCH_2_*CH_2_*CH_2_), 1.25 (m, 6H, CH_2_*CH_2_*CH_2_), 0.88 (m, 8H, SiC*H*_2_CH_2_S), 0.57 (m, 10H, SiC*H*_2_), 0.00 (s, 6H, Si(C*H*_3_)_2_), −0.09 (s, 3H, SiC*H*_3_). ^13^C[3]-NMR (CDCl_3_): δ (ppm) 184.2 (N*C*N), 121.9, 120.8 (N*C*H*C*HN), 59.2 (*CH_2_*N(CH_3_)_2_), 51.6 (NC*H*_2_), 45.5 (N(C*H*_3_)_2_), 38.8 (NC*H*_3_), 35.4 (NCH_2_*CH_2_*CH_2_), 29.9, 27.8 (S*CH_2_*), 21.0–13.6 (Si*CH_2_CH_2_*), −5.1, −5.2 (SiC*H*_3_). Elemental analysis calculated (%) for C_41_H_91_AgClN_6_S_4_Si_3_ (1024.03): C 48.09, H 8.96, N 8.21, S 12.52; found C 47.89, H 8.42, N 7.66, S 10.85.

#### 2.2.2. Synthesis of AgClMesImidG_2_(S(CH_2_)_2_NMe_2_)_4_ (**7**)

AgClMesImidG_2_(S(CH_2_)_2_NMe_2_)_4_ (**7**) was obtained by the same method as that for **1**, using as reagents compound MesImidG_2_(S(CH_2_)_2_NMe_2_)_4_ (**VII**) (50 mg, 0.05 mmol) and Ag_2_O (11.4 mg, 0.05 mmol). Compound **7** was obtained as brown oil (53 mg, 95%). ^1^H-NMR (CDCl_3_): δ (ppm) 7.24, 7.16 (m, 2H, NC*H*C*H*N), 6.95 (s, 2H, C*H_arom._*), 4.20 (t, 2H, J = 7.0 Hz, NC*H*_2_), 2.61–2.46 (m, 24H, *CH_2_*S*CH_2_CH_2_*N), 2.30 (s, 3H, CH_3*arom*_.), 2.22 (s, 24H, N(*CH_3_*)_2_), 1.94 (s, 8H, CH_3*arom.*_ y NCH_2_*CH_2_*CH_2_), 1.29 (m, 6H, CH_2_*CH_2_*CH_2_), 0.88 (m, 8H, SiC*H*_2_CH_2_S), 0.52 (m, 10H, SiC*H*_2_), -0.00 (s, 6H, Si(C*H*_3_)_2_), –0.10 (s, 3H, SiC*H*_3_). ^13^C[3]-NMR (CDCl_3_): δ (ppm) 181.2 (N*C*N), 139.5–134.9 (*C_ipso_*), 129.6 (*C*H*_arom._*), 122.9, 120.8 (N*C*H*C*HN), 59.4 (*C*H*_2_*N(CH_3_)_2_), 52.0 (N*C*H_2_), 45.6 (N(C*H*_3_)_2_), 35.6 (NCH_2_*C*H*_2_*CH_2_), 30.1, 28.9 (S*C*H*_2_*), 21.2 (*C*H_3*arom. para*_) 18.8–13.7 (Si*CH_2_CH_2_* y CH_3*arom. ortho*_), −5.0 (SiC*H*_3_). Elemental analysis calculated (%) for C_49_H_99_AgClN_6_S_4_Si_3_ (1128.19): C 52.17, H 8.85, N 7.45, S 11.37; found C 51.50, H 8.40, N 7.68, S 9.32.

### 2.3. Synthesis of Imidazolium-Terminated Bow-Tie and Precursor Dendrimers

#### 2.3.1. Synthesis of (Cl(CH_3_)_2_Si)_2_G_1_[OC_6_H_4_O]G_1_(Si(CH_3_)_2_Cl)_2_ (**8**)

To the compound (A_2_G_1_[OC_6_H_4_O]G_1_A_2_) (**VIII**) (0.2 g, 0.4 mmol, A denotes allyl), previously synthetized in our research group [21], HSi(CH_3_)_2_Cl (0.5 mL, 4.6 mmol) and Karstedt catalyst were added and heated at 60°C for 4h. Then, all volatiles were removed, giving compound **8** as a yellowish oil, and used for the next reaction step without further purifications (0.350 g, 98%). Purification was carried out on the final compound **11**. ^1^H-NMR (CDCl_3_): δ (ppm) 6.80 (s, 4H, OC_6_*H*_4_O), 3.89 (t, 4H, J = 6.4 Hz, OC*H*_2_), 1.74 (m, 4H, OCH_2_C*H*_2_), 1.41 (m, 12H, CH_2_C*H_2_*CH*_2_*), 0.89, 0.59 (m, 20H, C*H*_2_Si), 0.38 (s, 24H, C*H*_3_SiCl), −0.05 (s, 6H, C*H*_3_Si).

#### 2.3.2. Synthesis of (H(CH_3_)_2_Si)_2_G_1_[OC_6_H_4_O]G_1_(Si(CH_3_)_2_H)_2_ (**9**)

To a solution of **8** (0.350 g, 0.2 mmol) in dry diethyl ether, LiAlH_4_ 2.4M (0.5 mL) was added in cold addition and drop by drop and the mixture stirred for 16 h at room temperature. Then, extraction in H_2_O/diethyl ether was carried out and removal of the volatiles from the organic phase afforded **9** as colorless oil (87 mg, 55%). Compound **9** was used in the next reaction step without further purification. Purification was carried out on the final compound **11**. ^1^H-NMR (CDCl_3_): δ (ppm) 6.80 (s, 4H, OC_6_*H*_4_O), 3.89 (t, 4H, J = 6.4 Hz, OC*H*_2_), 3.83 (m, 4H, (CH_3_)_2_Si*H*), 1.75 (m, 4H, OCH_2_C*H*_2_), 1.49–1.31 (m, 12H, CH_2_C*H_2_*CH*_2_*), 0.64, 0.49 (m, 20H, C*H*_2_Si), 0.04 (d, 4H, J = 3.7 Hz, (C*H*_3_)_2_SiH), −0.08 (s, 6H, C*H*_3_Si).

#### 2.3.3. Synthesis of (Br(CH_2_)_4_Si)_2_G_1_[OC_6_H_4_O]G_1_(Si(CH_2_)_4_Br)_2_ (**10**)

The next step consisted in the addition of 1-bromobutane (55 µl, 0.54 mmol) and Karstedt catalyst to compound **9** (0.087 g, 0.12 mmol) and the mixture was stirred for 16h at 60°C. Compound **10** was obtained as a brown oil after filtering to remove catalyst traces and evaporating all volatiles (140 mg, 93%). It was used for the next reaction step without further purifications. Purification was carried out on the final compound **11**. ^1^H-NMR (CDCl_3_): δ (ppm) 6.80 (s, 4H, OC_6_*H*_4_O), 3.88 (t, 4H, J = 6.4 Hz, OC*H*_2_), 3.40 (t, 8H, J = 6.8 Hz, CH_2_Br), 1.85, 1.75 (m, 12H, OCH_2_C*H*_2_ and BrCH_2_C*H*_2_), 1.49–1.23 (m, 20H, CH_2_C*H_2_*CH*_2_*), 0.61–0.43 (m, 20H, C*H*_2_Si), −0.06 (s, 24H, C*H*_3_Si), −0.08 (s, 6H, C*H*_3_Si).

#### 2.3.4. Synthesis of (BrMeImid(CH_2_)_4_Si)_2_G_1_[OC_6_H_4_O]G_1_(Si(CH_2_)_4_ImidMeBr)_2_ (**11**)

To a solution of **10** (140 mg, 0.12 mmol) in dry acetone, 1-methylimidazole (0.04 mL, 0.5 mmol) was added and the mixture stirred for 24h at 90°C. For purification purposes, a dialysis membrane (Spectra/Por Dialysis Membrane, Biotech Ce Tubing, MWCO:100-500 D, New Jersey, USA) was used for three days in distilled water. The first-generation bow-tie was obtained as a yellowish oil (129 mg, 68%). ^1^H-NMR (CD_3_OD): δ (ppm) 8.94 (s, 4H, NC*H*N), 7.63, 7.58 (s, 8H, NC*H*C*H*N), 6.81 (s, 4H, OC_6_*H*_4_O), 4.24 (t, 8H, J = 7.4 Hz, NC*H*_2_), 3,97–3.93 (m, 16H, C*H*_3_N and OC*H*_2_), 1.89 (m, 8H, NCH_2_C*H*_2_), 1.78 (m, 4H, OCH_2_C*H*_2_), 1.37 (m, 20H, CH_2_C*H_2_*CH*_2_*), 0.60 (m, 20H, C*H*_2_Si), −0.03 (s, 24H, C*H*_3_Si), −0.05 (s, 6H, C*H*_3_Si). ^13^C[3]-NMR (CD_3_OD): δ (ppm) 154.6 (*C_ipso_*), 124,9 and 123.6 (N*C*H*C*HN), 116.5 (*C*H*_arom_*.), 69.2 (O*C*H_2_), 50.5 (N*C*H_2_), 36.5 (*C*H_3_N), 34.9 (NCH_2_*C*H_2_), 21.9–14,7 (Si*C*H_2_*C*H_2_), −3.2 and −4.7 (*C*H_3_Si). Elemental analysis calculated (%) for C_68_H_130_Br_4_N_8_O_2_Si_6_ (1579.97): C 51.69, H 8.29, N 7.09; found C 52.47, H 8.81, N 7.49. ESI-TOF [M-Br]^+^ = 1500.65.

#### 2.3.5. Synthesis of (Cl(CH_3_)_2_Si)_4_G_2_[OC_6_H_4_O]G_2_(Si(CH_3_)_2_Cl)_4_ (**12**)

The procedure to obtain **12** was the same as the one used for **8**_,_ although this time A_4_G_2_[OC_6_H_4_O]G_2_A_4_ (**IX**) (475 mg, 0.5 mmol, A denotes allyl) [21] and HSi(CH_3_)_2_Cl (0.523 mL, 4.8 mmol) were used to afford **12** as yellow oil and used for the next reaction step without further purifications (0.84 mg, 97%). Purification was carried out on the final compound **15**. ^1^H-NMR (CDCl_3_): δ (ppm) 6.80 (s, 4H, OC_6_*H*_4_O), 3.89 (t, 4H, J = 6.4 Hz, OC*H*_2_), 1.74 (m, 4H, OCH_2_C*H*_2_), 1.37 (m, 28H, CH_2_C*H_2_*CH*_2_*), 0.84, 0.51 (m, 36H, C*H*_2_Si), 0.37 (s, 48H, C*H*_3_SiCl), −0.07 (s, 18H, C*H*_3_Si).

#### 2.3.6. Synthesis of (H(CH_3_)_2_Si)_4_G_2_[OC_6_H_4_O]G_2_(Si(CH_3_)_2_H)_4_ (**13**)

The compound was obtained using the same procedure as for **9**, adding **12** (0.84 mg, 0.5 mmol) and LiAlH_4_ 2.4M (2 mL) to afford **13** as a yellowish oil and used for the next reaction step without further purifications (720 mg, 99%). Purification was carried out on the final compound **15**. ^1^H-NMR (CDCl_3_): δ (ppm) 6.80 (s, 4H, OC_6_*H*_4_O), 3.89 (t, 4H, J = 6.4 Hz, OC*H*_2_), 3.82 (m, 8H, (CH_3_)_2_Si*H*), 1.75 (m, 4H, OCH_2_C*H*_2_), 1.50–1.20 (m, 28H, CH_2_C*H_2_*CH*_2_*), 0.62, 0.50 (m, 52H, C*H*_2_Si), 0.04 (d, 4H, J = 3.7 Hz, (C*H*_3_)_2_SiH), −0.08 (s, 18H, C*H*_3_Si).

#### 2.3.7. Synthesis of (Br(CH_2_)_4_Si)_4_G_2_[OC_6_H_4_O]G_2_(Si(CH_2_)_4_Br)_4_ (**14**)

The procedure to synthesize this compound was the same as that to obtain **10**, adding **13** (720 mg, 0.5 mmol) and 1-bromobutane (4.4 mmol, 0.45 mL). Compound **14** was obtained as a brown oil (1.13 g, 89%). It was used for the next reaction step without further purifications. Purification was carried out on the final compound **15**. ^1^H-NMR (CDCl_3_): δ (ppm) 6.78 (s, 4H, OC_6_*H*_4_O), 3.86 (t, 4H, J = 6.4 Hz, OC*H*_2_), 3.41 (t, 16H, J = 6.8 Hz, CH_2_Br), 1.86 (m, 20H, OCH_2_C*H*_2_ and BrCH_2_C*H*_2_), 1.41–1.29 (m, 44H, CH_2_C*H_2_*CH*_2_*), 0.56–0.42 (m, 68H, C*H*_2_Si), −0.01 (s, 6H, C*H*_3_Si), −0.09 (s, 48H, C*H*_3_Si), −0.10 (s, 12H, C*H*_3_Si).

#### 2.3.8. Synthesis of (BrMeImid(CH_2_)_4_Si)_4_G_2_[OC_6_H_4_O]G_2_(Si(CH_2_)_4_ImidMeBr)_4_ (**15**)

The titled compound was prepared as described for **11**_,_ using **14** (1.13 g, 0.4 mmol) and 1-methylimidazole (0.23 mL, 3.2 mmol) to afford compound **15** as a brown oil (0.93 mg, 73%). For purification of compound **15**, a dialysis membrane (Spectra/Por Dialysis Membrane, Biotech Ce Tubing, MWCO:100-500 D) was used for three days in distilled water. ^1^H-NMR (CD_3_OD): δ (ppm) 9.04 (s, 8H, NC*H*N), 7.65, 7.59 (s, 16H, NC*H*C*H*N), 6.81 (s, 4H, OC_6_*H*_4_O), 4.24 (t, 16H, J = 7.3 Hz, NC*H*_2_), 3.94 (m, 28H, C*H*_3_N and OC*H*_2_), 1.89 (m, 16H, NCH_2_C*H*_2_), 1.76 (m, 4H, OCH_2_C*H*_2_), 1.38 (m, 44H, CH_2_C*H_2_*CH*_2_*), 0.60 (m, 68H, C*H*_2_Si), 0.06 (s, 6H, C*H*_3_Si), −0.01 (s, 48H, C*H*_3_Si), −0.06 (s, 12H, C*H*_3_Si). ^13^C[3]-NMR (CD_3_OD): δ (ppm) 154.5 (*C_ipso_*), 124,9 and 123.6 (N*C*H*C*HN), 116.6 (*C*H*_arom_*.), 69.3 (O*C*H_2_), 50.5 (N*C*H_2_), 36.5 (*C*H_3_N), 34.9 (NCH_2_*C*H_2_), 24.9–15.8 (Si*C*H_2_*C*H_2_), 1.05 and −4.4 (*C*H_3_Si). Elemental analysis calculated (%) for C_136_H_270_Br_8_N_16_O_2_Si_14_ (3194.19): C 51.14, H 8.52, N 7.02; found C 51.09, H 8.72, N 7.94. ESI-TOF: no assignable peaks were detected.

### 2.4. Antibacterial Experiments

#### 2.4.1. Dendritic and Metallodendritic Systems

Two different families of dendritic systems were studied. The first one was based on cationic imidazolium salts of different topologies (spherical dendrimers **I**–**V**, dendrons **VI**–**VII** and bow-ties **11** and **15**). The second family consisted on metallodendrimers with spherical and dendron topology including Ag(I)-NHC in their structures (spherical dendrimers **1**–**5**, dendrons **6**–**7**). All these compounds were chosen in order to compare sizes, topologies, and the influence of a metal in the structure.

Serial dilutions for the imidazolium salt systems in sterile distilled water were used to perform the biological assays. The solubility in aqueous medium for the mesitylimidazolium salts and metallodendrimers was enhanced by adding dimethyl sulfoxide (DMSO) 1%.

#### 2.4.2. Bacteria Strains and General Growth Conditions

For the different assays, all the bacterial strains used are listed in Table 1. *E. coli, S. aureus*, and *B. subtilis* were routinely grown in Mueller Hinton (MH) medium at 37 °C and 220 r.p.m. Subsequently, *E. coli* and *S. aureus* were diluted until *OD*_600_ between 0.08 and 0.11 AU (absorbance units), whereas *B. subtilis* was freshly diluted and incubated for 2–3 h more until bacteria reached the exponential phase.

#### 2.4.3. MIC and MBC Assays

The protocol was based on the ISO 20776-1:2006 standards [34]. Once the microorganisms were inoculated in MH at 37 °C and 220 r.p.m., they was incubated with the biocides in sterile 96-well plates at different concentrations. As a negative control, Muller-Hinton (MH) media was used, in order to exclude any contamination, and as a positive control, the inoculum (sample without biocide) was used to check the proper growth of bacteria. Additionally, the correct growth of microorganism was checked, using as control 1% of DMSO, because some samples were not completely water soluble. Plates were analyzed at 24 h at 37 °C using a wavelength of 600 nm. An Ultra Microplate reader was used (BIO-TEK Instruments, model ELx808, Winooski, VT, United States) for *E. coli* and *S. aureus*, and a Multi-Mode Microplate Reader (BIO-TEK Synergy 2, Winooski, VT, USA) for *B. subtilis*. Evaluating the results, the concentration used where no bacterial growth was observed indicated the minimum inhibitory concentration (*MIC*) of the compound. In order to determine the minimum bactericidal concentration (*MBC*) values, 5 µL of each sample were taken and deposited on a Petri dish with Plate Account Agar (PCA) in the case of *E. coli* and *S. aureus*, and a Petri dish with Mueller Hinton Agar for *B. subtilis*. Finally, they were incubated for 24 h at 37 °C.

### 2.5. Biosensors Assay

The biosensor assay was performed as described previously [35,36]. In brief, overnight cultures were grown in MH medium, and supplemented with respective antibiotics for selection. On the next day, cells were re-inoculated and grown till the exponential phase (~*OD*_600_ 0.2). Subsequently, the biosensor strains [27] (P*_liaI_*, P*_fabHB_*, P*_yxdL_*, P*_bceA_*, P*_pspA_*, and P*_psdA_*) were diluted to an *OD*_600_ 0.01. Then, 100 µL of cells per sample were transferred to a 96-well plate (black walls, clear bottom, Greiner Bio-One, Frickenhausen, Germany). Growth and luminescence were obtained every five minutes in a Synergy^TM^ NeoalphaB 2 plate reader (BioTek, Winooski, VT, USA) controlled by Gen 5 software. After one hour, compounds of interest were added at indicated concentrations (close to *MIC* values) and strains were monitored for an additional 4h. For the non-induced control, an equivalent volume of MH was added. For some biosensor strains, the antimicrobial peptide bacitracin (final concentration of 30 µg/mL) was added as a positive control. Results were processed and figures generated by applying GraphPad Prism 6 (free software, San Diego, CA, USA, 2012).

### 2.6. Mode of Action: Cell Membrane Depolarization

To test if compounds of interest interfere with the cell membrane polarization state of *B. subtilis* cells, a depolarization assay based on the voltage sensitive dye Disc_3_(5) was applied [33]. In brief, *B. subtilis* cells were cultivated as described above and transferred into a 96-well plate (black walls, clear bottom, Greiner Bio-One, Frickenhausen, Germany). The autofluorescence of *B. subtilis* was recorded at 610 (excitation) and 660 nm (emission) for three minutes using a Synergy^TM^ NeoalphaB 2 plate reader (BioTek, Winooski, VT, USA) run by Gen5 software. After, Disc_3_(5) (1 µM, final concentration) was added and the incorporation of the dye into the cell membrane was monitored for another seven minutes until steady fluorescence levels were reached. Subsequently, compounds of interest were added to the indicated concentrations and changes of fluorescence were obtained for an additional hour at one-minute intervals. To reject any possibility of an interaction between the dye and the respective compounds, the fluorescence of MH containing Disc_3_(5) [33] and BSA (0.5 mg/mL) was also measured in the absence of bacteria. BSA was added to reduce the absorption of the applied dyes to polystyrene surfaces of the microtiter plates. As controls, the antibiotic gramicidin (5 µM, final concentration), 1% DMSO, and water were used. The obtained data were processed to generate final graphs using GraphPad Prism 6.

### 2.7. Mode of Action: Cell Membrane Perturbation

To further characterize the effects of cell membrane depolarization for potential cell membrane perturbation, the procedure described above was extended by applying the DNA-binding dye Sytox Green [37]. For this, *B. subtilis* was grown as described above. Cells were again diluted to on *OD*_600_ 0.2 supplemented with BSA (0.5 mg/mL). Subsequently, 200 µL were transferred to a 2-mL reaction tube and mixed with Sytox Green (50 nM, final concentration) and Disc_3_(5) (1 µM final concentration). The reaction tube was incubated for five minutes in the Eppendorf Thermomixer comfort at 37 °C and 1000 r.p.m with the lid open, to allow sufficient aeration. Addition of the compounds of interest for 10 min was carried out right after at indicated concentrations and 2 µL were transferred to an agarose pad (1% UltraPure Agarose pads, Invitrogen, Waltham, MA, USA). As a positive control of pore formation, it was added the antibacterial peptide Nisin (5 µM, final concentration) and, as a negative control, sterile distilled water. To exclude the interference of dyes and compounds, the assay was performed in the absence of cells. Fluorescence microscopy was carried out using a ZEISS Axio 7 inverse microscope (Carl Zeiss, Jena, Germany run by Zen 2.3 Pro software, 2016) equipped with standard Cye5 (Ex: 650/Em: 673) and eGFP (Ex: 488/Em: 509) filter sets. All pictures were processed using the ImageJ-Fiji program (free software) [38] equipped with the plugin MicrobeJ [39]. For each compound tested, at least 200 cells were marked as the region of interest. To quantify the effects of depolarization and cell membrane perturbation, the mean pixel intensities of both channels (Cye5 and eGFP) were obtained.

### 2.8. Mode of Action: Nile Red Assay

In a 2-mL Eppendorf, the working solution of *B. subtillis* WT168 (*OD*_600_ 0.02) was mixed with the samples (with the concentrations used in the previous assay) in an Eppendorf Thermomixer comfort at 37ºC, and at 1000 r.p.m for 1 min with the lid open. Then, Nile red (1 µg/mL) was added and placed in UltraPure Agarose pads (Invitrogen) previously prepared. A ZEISS Axio Observer Microscope (Zen 2.3 Pro software) with Axiocam 702 mono was used to visualize the samples at 511 nm. The procedure was repeated but with incubation for 20 min. All pictures were processed using ImageJ-Fiji program [38] equipped with the plugin MicrobeJ [39]. As controls, sterile distilled water and sterile DMSO 1% were used.

### 2.9. Mode of Action: Protein Delocalization Assay

From overnight culture, the strains were incubated for 2–3 h at 37 °C and 220 r.p.m supplemented with xylose as indicated. For MinD, 0.1% and for MreB, 0.3% xylose (final concertation) were added. In a 2-mL Eppendorf, *OD*_600_ 0.2 cell solution of *B. subtilis* and compounds (at concentration depicted above) were mixed, and they were incubated in an Eppendorf Thermomixer at 37 °C and 1000 r.p.m, for 1 (or 20, as indicated) minute(s) with the lid open to allow full aeration. Then, 3 µL of cells were transferred onto UltraPure (Invitrogen) agarose pads and microscopy was performed using an Axio Observer 7 inverse microscope (Carl Zeiss, Jena, Germany) run by the Zen 2.3 Pro software. Fluorescence signals were obtained using standard eGFP (Ex: 488/EM 509) filter sets. Untreated cells and cells exposed to 1% DMSO (final concertation) were included and serve as controls. Image processing was performed using the ImageJ-Fiji program [38] equipped with the plugin MicrobeJ [39].

## 3. Results and Discussion

### 3.1. Synthesis of the Dendritic Family Containing Ag(I)-NHC

**Scheme 1:**
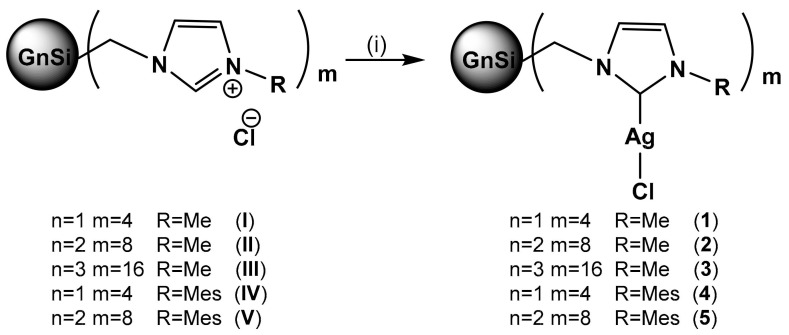
Synthetic route for compounds **1**–**5**: (i) Ag_2_O, CH_2_Cl_2_, 8 h.

The synthesis of dendritic systems containing Ag(I)-NHC in the periphery of a spherical dendrimer or at the focal point of a dendron was carried out using as precursors the family of cationic dendritic systems with imidazolium salts (**I**–**VII**) described in one of our previous works [28] (see Supplementary Material Figures S1 and S2). Roman numbers were used for already reported compounds while Arabic numbers for the new ones. Ag_2_O was used as metalating agent but also taking advantage of its basicity to generate the carbene fragments in situ (Scheme 1) [40]. The formation of the desired spherical dendrimers **1**–**5** was easily assessed by ^1^H-NMR spectroscopy, due to the disappearance of the singlet of the imidazolium proton at *c.a.* 9 ppm. In the ^13^C{^1^H}-NMR spectroscopy, there was also a shift from 137 to *c.a.* 180 ppm of the carbon atom at the imidazolium salt signal once the carbene atom was formed, confirming the formation of the Ag(I)-NHC fragment (**1–5**) (see Supplementary Material Figures S3 and S4).

**Scheme 2:**
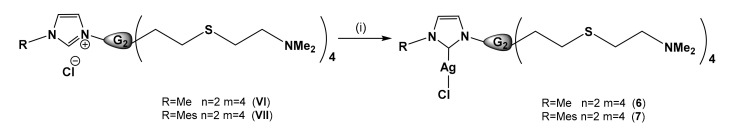
Synthetic route for compounds **6**–**7**: (i) Ag_2_O, CH_2_Cl_2_, 8 h.

Regarding the dendrons, the incorporation of the metal fragment in compounds **6**–**7** was obtained using the same protocol (Scheme 2), observing in ^1^H-NMR spectroscopy the disappearance of the singlet signal of NC*H*N at 10.5 ppm, and confirming the formation of the carbene bond. In the same way, by ^13^C{^1^H}-NMR spectroscopy, the signal at 140 ppm associated to N*C*HN disappeared and that of the carbene–metal bond formation observed at 184-181 ppm (see Supplementary Material Figures S5 and S6).

The stability of Ag(I)-NHC compounds in water was monitored by following the changes of the NHC ligand in the ^1^H-NMR spectra in a mixture 1:1 (D_2_O/DMSO-d_6_). For compounds **1**–**3** containing an Me group in the NHC fragment, minor degradation of *c.a*. 10% or 15% was observed at 2 and 8 h, respectively, without significant differences respecting the generation. However, at 24 h, such differences between compounds became more visible. For compound **1**, the decomposition was c.a. 20% while second (**2**) and third (**3**) generations degraded around 50% and 65%, respectively. The degradation process consisted of the formation of the precursors containing imidazolium groups in the dendritic structure, according to the ^1^H-NMR spectra and reported elsewhere [28]. In the case of compound **4** and **5** with a mesityl group at the NHC ligand, no degradation was detected for at least 48 h (see Supplementary Material Figures S7–S10). The stability of the NHC-Ag(I) complexes strongly depends on the nature of the substituents on the NHC fragment. For sterically unhindered monometallic Ag(I)-NHC bearing saturated substituents and reported elsewhere [41], hydrolysis was found to be fast (95% degradation at 2 h). This data contrast with those observed for the systems **1**–**3**, where the dendritic scaffold must play a role in certain stabilization. On the other hand, monometallic Ag(I)-NHCs bearing unsaturated and aromatic substituents were resistant to decomposition, only observing degradation upon heating [42]. For dendrons **6** and **7**, the same tendency was observed. Dendron **7**, bearing a mesityl group, seemed to be more stable than the one with a methyl group (**6**), reaching 15% and 70% at 2 h and 50% and 80% after 24 h, respectively, of degradation (see Supplementary Material Figures S11–S12). However, in terms of topology, spherical dendrimers were more stable than dendrons.

The water stability study must be taken into account when analyzing the microbiological experiments. For MIC and MBC studies, 24 h were needed to perform the assays and therefore different degrees of hydrolyzed NHC branches may occur in the whole dendritic molecule. However, for the biosensors and mode of action assays, where 5 h or minutes were needed, respectively, the integrity of the dendritic Ag(I)-NHC molecule must be considered (vide infra).

### 3.2. Synthesis of Bow-Ties with Methylimidazolium Salts in the Periphery

**Scheme 3:**
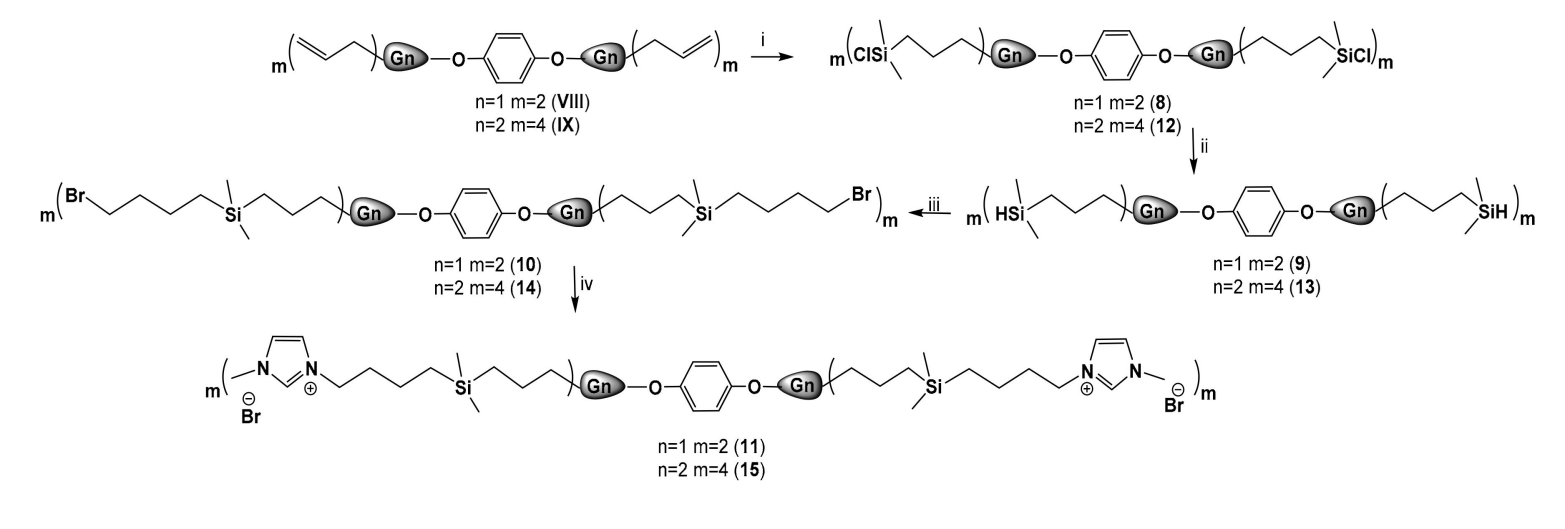
Synthetic pathway of **11** and **15**: (**i**) HSiMe_2_Cl, [Pt], 60 °C, 4 h; (**ii**) LiAlH_4_, diethyl ether, 16 h; (**iii**) 1-bromobutane, [Pt], 60 °C, 16 h; (**iv**) 1-methylimidazole, acetone, 90 °C, 24 h.

For comparative purposes, two different generations of bow-ties with imidazolium salts in the periphery (see Supplementary Material Figure S13) were synthesized using a similar synthetic route described previously in our research group [21] (Scheme 3). The first step consisted of an hydrolyzation reaction of (A*_m_*G*_n_*[OC_6_H_4_O]G*_n_*A*_m_* (*n* = 1, *m* = 2 (**VIII**); *n* = 2, *m* = 4 (**IX**)) with HSi(CH_3_)_2_Cl [36], affording the compounds (Cl(CH_3_)_2_Si)_m_G_n_[OC_6_H_4_O]G_n_(Si(CH_3_)_2_Cl)_m_ (where *n* = 1, *m* = 2 (**8**); *n* = 2, *m* = 4 (**12**)). For these derivatives, the most relevant signal in the ^1^H-NMR spectroscopy is the singlet at 0.38 ppm attributed to the -SiClMe_2_ group and the corresponding disappearance of the signals of the allyl groups around 5–6 ppm. The second step consisted in the treatment of **8** or **12** with LiAlH_4_ as reducing agent to afford (H(CH_3_)_2_Si)_m_G_n_[OC_6_H_4_O]G_n_(Si(CH_3_)_2_H)_m_ (where *n* = 1, *m* = 2 (**9**); *n* = 2, *m* = 4 (**13**)) [43]. The reaction was followed by ^1^H-NMR spectroscopy, observing the disappearance of the singlet due to -SiMe_2_Cl at 0.40 ppm and formation of a new doublet signal at 0.04 ppm and a multiplet at 3.80 ppm, both due to the fragment -SiMe_2_H. Then, another hydrolyzation using compound **9** or **13** over four or eight equivalents of 1-bromobutane, respectively, produced the compounds (Br(CH_2_)_4_Si)_m_G_n_[OC_6_H_4_O]G_n_(Si(CH_2_)_4_Br)_m_ (where *n* = 1, *m* = 2 (**10**); *n* = 2, *m* = 4 (**14**)). The reactions were followed by disappearance of the signals corresponding to the -SiMe_2_H grouping. The last step was the introduction of 1-methylimidazole by replacing the terminal bromine atom. In this case, the bromine-terminated dendrimers were used as platform to quaternize the imidazole ring, affording the desired imidazolium-terminated dendrimers (BrMeImidButSi)_m_G_n_[OC_6_H_4_O]G_n_(SiButImidMeBr)_m_ (*n* = 1, *m* = 2 (**11**); *n* = 2, *m* = 4 (**15**)). ^1^H-NMR spectroscopy evidenced the shifting of the triplet signal around 3.4 ppm of the -C*H*_2_Br group to 4.20 ppm once the imidazolium group was included, obtaining **11** and **15** where the presence of signals attributed to the Me-imidazolium were assigned (see Supplementary Material Figure S14–S22).

### 3.3. Microbiological Experiments

#### MIC and MBC Assay

The MIC (minimal inhibitory concentration) and MBC (minimal bactericide concentration) assays were performed against two types of Gram-positive bacteria (*S. aureus* and *B. subtilis*) and one type of Gram-negative bacteria (*E. coli*). Different topologies of imidazolium-containing carbosilane dendritic systems (**I–VII**, **11**, and **15**) were used in order to study the influence of their shape in respect to their antibacterial activity (see Table 2). In addition, to evaluate the presence of silver(I) in the dendritic systems (**1–7**), a comparative study with the cationic dendritic imidazolium salts was carried out (see Table 3). Some compounds, mainly those containing mesityl imidazolium groups, required 1% of DMSO as solvent to be dissolved. Nevertheless, it was checked that 1% DMSO did not cause any antibacterial activity.

All cationic imidazolium systems **I**–**VII**, **11**, and **15** presented promising bactericidal activity, resulting in low values of MIC and MBC (Table 2). The activity seemed to be a compromise between different parameters, like shape, number of positive charges, generation, hydrophobicity, and nature of the imidazolium groups, that make rationalization difficult.

For example, comparing first-generation dendrimers **I** and **11,** both having four positive imidazolium groups but different shapes, the bow-tie system showed in general enhanced antibacterial properties. However, the opposite behavior was observed for the analogous second-generation dendrimers **II** and **15**. In this context, the different counter-anion used in both topologies cannot explain the contrary behaviors observed in bow-ties and spherical dendrimers. Comparing the imidazolium substituents in the spherical dendrimers **I** and **IV**, it becomes apparent that the mesityl derivative was more active than the methyl, although this behavior was lost for the second generations **II** and **V**. For dendrons **VI** and **VII,** the antibacterial behavior was close to the first generation bow-tie **11** or to the spherical second-generation dendrimer **II**, suggesting that their antibacterial activity did not only depend on the nature of the imidazolium group, but rather on the presence of amine groups probably protonated in aqueous medium. Therefore, the lipophilic-hydrophilic balance within the molecules imposed by the different parameters introduced must be a decisive factor for their antibacterial activity.

The MIC and MBC values of Ag(I)-NHC compounds against the three different bacteria are summarized in Table 3. In all cases, effective bactericidal activity was observed to be higher than that detected for monometallic compounds described elsewhere [44].

In general, the presence of metal in the dendritic structure did not lead to a significant improvement in the antibacterial activity compared with their metal-free cationic precursors. However, it is worthwhile highlighting that the presence of silver(I) played an important role in compound **1**, resulting in an increased susceptibility of *E. coli* in both MIC and MBC, compared to the cationic precursor **I**. Interestingly, the presence of AgNO_3_ was reflected in lower MIC and MBC values than dendrimers; however, comparing the activity of AgNO_3_ with the silver dendrimers **1**–**7** in molar ratio or per silver atom (see Supplementary Material Table S1), no significant differences in the activity were observed.

In order to understand the mode of action of Ag(I)-containing and imidazolium-terminated dendritic systems trying to visualize possible differences between them, several bioassays were carried out and are shown in the following sections.

### 3.4. Biosensors Assay

Whole-cell biosensors are a convenient and powerful screening tool in antibiotic research to narrow down the cellular target of antimicrobial action [27,45]. Six different *Bacillus subtilis* biosensors (see Experimental Section, Table 1) were used to visualize the response of a reporter strain to different aspects of envelope stress caused by the presence of the dendritic systems. Inducing concentrations close to but below the *MIC* values were chosen to avoid bacterial death. The optical density (*OD*_600_) and luminescence read-outs were measured at the same time to monitor the growth and biosensor response of the treated reporter strains, using bacitracin-treated (Bac; final concentration 30 µg/mL) and -untreated samples as a positive and negative control, respectively. The 1% DMSO control was not included, as it did not affect the proper cell growth in the previous experiment.

The dendritic systems with imidazolium salts (**I–VII** and **11** and **15**) and their analogy with Ag(I)-NHC (**1**–**7**) were used for this experiment. From the six-cell envelope-specific biosensors used, only P*_liaI_* and P*_psdA_* were induced by the Ag(I)-NHC dendritic systems, whereas the other biosensors gave no response (see Supplementary Material Figure S23).

**Figure 1:**
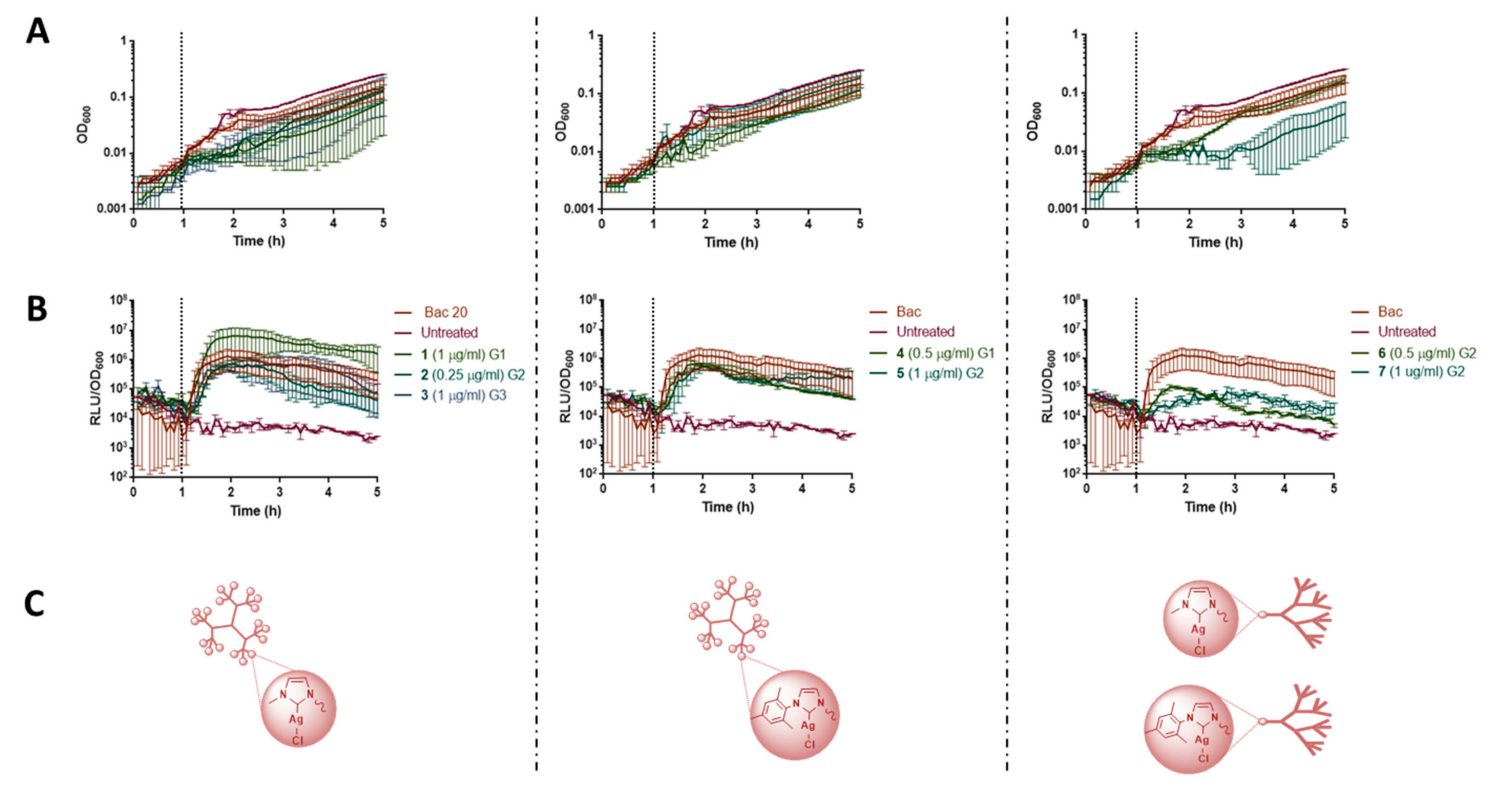
P*_liaI_* biosensors response in the presence of dendritic systems containing Ag(I)-NHC. (**A**) *OD*_600_ values; (**B**) relation between luminescence/*OD*_600_; (**C**) Schematic representation of compounds. Dash lines in graphs A and B at 1 h denote the addition of compound.

**Figure 2:**
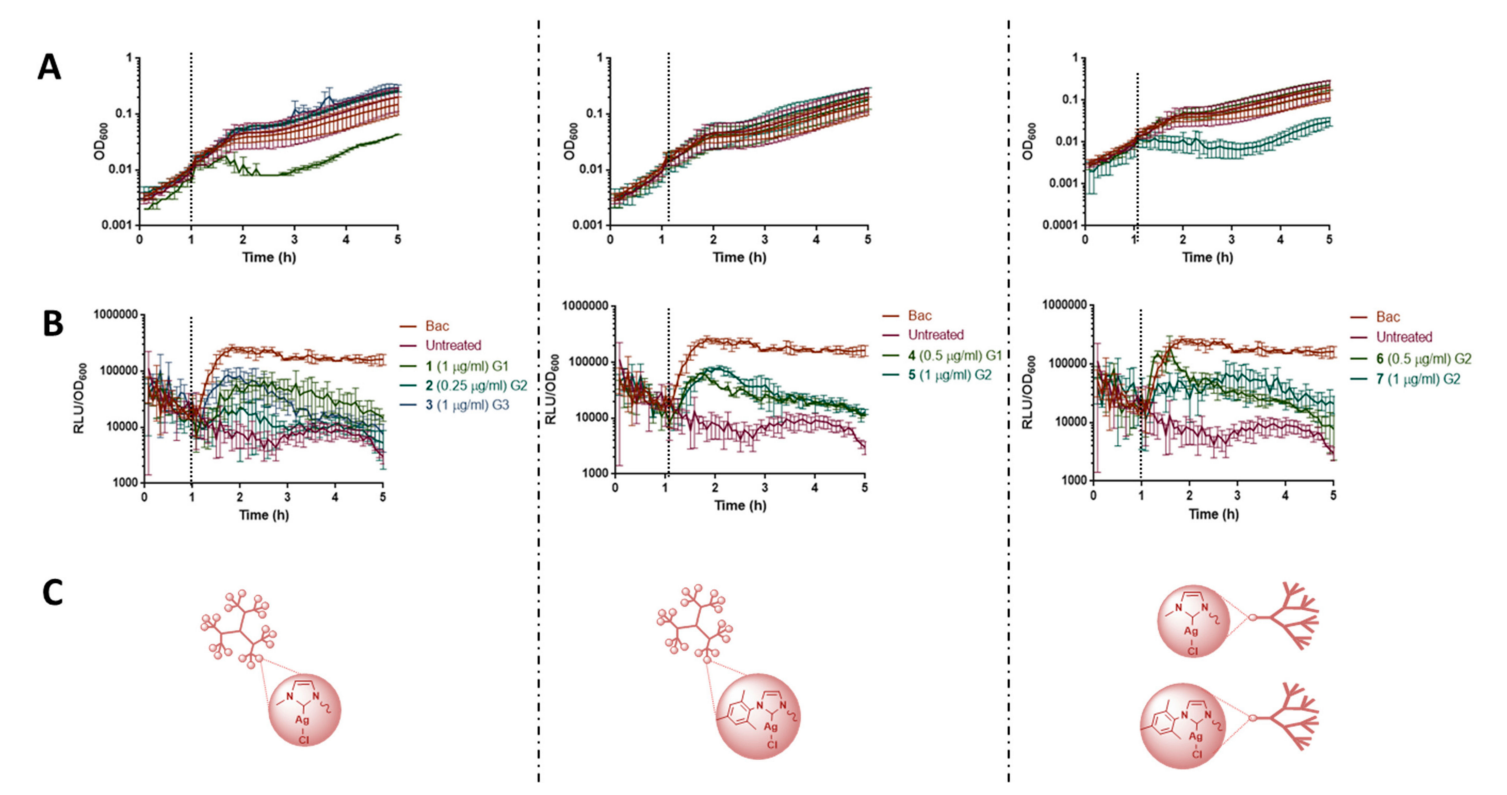
P*_PsdA_* biosensor induction in the presence of dendritic systems containing Ag(I)-NHC. (**A**) *OD*_600_ values; (**B**) relation between luminescence/*OD*_600_; (**C**) Schematic representation of compounds. Dash lines at 1h in graphs A and B denote the addition of compound.

For the P*_liaI_* biosensor (Figure 1), which responds to general cell envelope stress [45], the spherical Ag(I)-NHC dendrimers (**1**–**5**) showed a strong induction, which was even higher for **1** than for the positive control (Bac), while Ag(I)-NHC dendrons (**6**–**7**) triggered the P*_liaI_* response to a lesser extent. These results indicate that spherical dendrimers with a high silver content triggered a stronger envelope stress than dendrons that only contain one silver atom per molecule. It is possible that the polyvalency of the spherical dendrimers as well as their comparative water-stability respecting dendrons were important characteristics for this activation. With regard to the P*_psdA_* biosensor induction (see Figure 2), which has been described to respond to cell envelope stress caused by antimicrobial peptides’ response [46], the presence of compounds **1**–**7** led to moderate inductions, which was less correlated to the dendritic shape.

The use of AgNO_3_ salt (see Supplementary Material Figure S24A) did not induce any biosensor by itself, indicating that the organometallic fragment was needed for the corresponding envelope perturbation. Moreover, none of the cationic imidazolium-containing dendritic systems (**I–VII**, **11**, and **15**) were able to stimulate any biosensors (see Supplementary Material Figure S25), suggesting a different antibacterial mechanism of action than the Ag(I)-NHC dendritic derivatives.

It is tempting to postulate that the dendritic structures are crucial to facilitate the transport of silver atoms to their biological targets and that their subsequent release from the complex, which—based on their interference with the envelope—might be able to penetrate into the cytoplasm, ultimately disrupting the integrity of the bacterial cell. Based on the biosensor response and this hypothesis, we next aimed at analyzing the cell membrane-perturbating action of the compounds in more detail.

### 3.5. Mode of Action of Dendritic Systems at the Cell Membrane

In order to distinguish the possible modes of interference of these dendritic systems at the cell membrane, different complementary techniques were employed, using fluorescently labelled probes to investigate cell membrane depolarization (Disc_3_(5)), pore formation (Sytox Green), and alterations in cell membrane lipid composition (Nile red). Moreover, the delocalization of cell membrane-attached cytoskeletal proteins MinD and MreB was studied based on fluorescently labelled derivatives of these proteins.

### 3.6. Cell Membrane Depolarization: Fluorescence Assay (Disc_3_(5))

**Figure 3:**
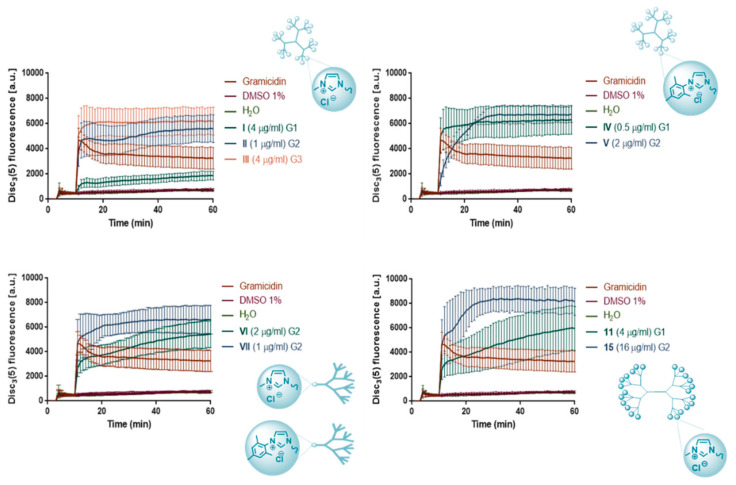
Disc_3_(5) fluorescence graphs cell membrane depolarization of *B. subtilis* WT168 in the presence of compounds I–VII, 11, and 15 with the corresponding schematic representation of compounds.

**Figure 4:**
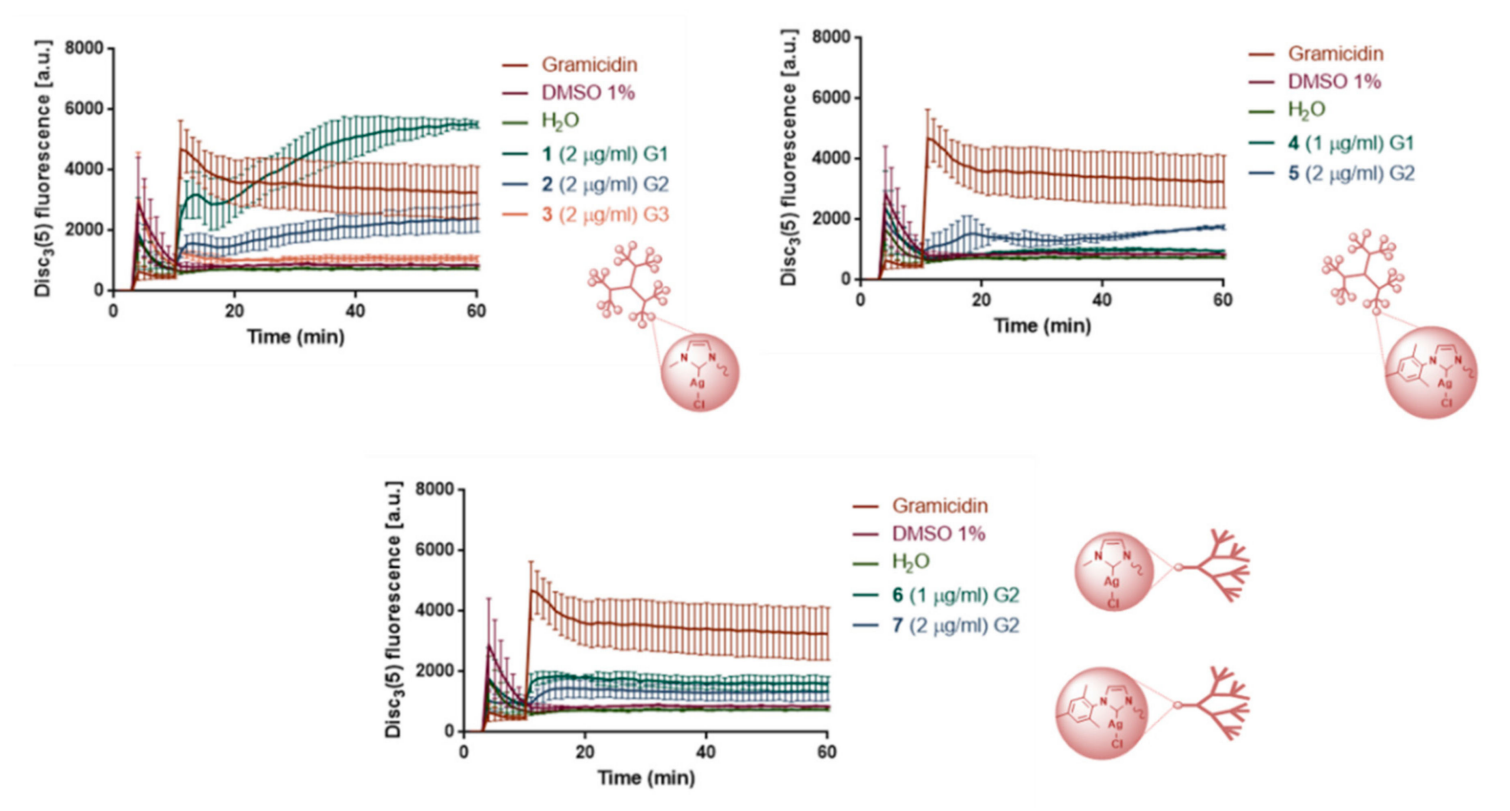
Disc_3_(5) fluorescence graphs cell membrane depolarization of *B. subtilis* WT168 in the presence of compounds 1–7 with the corresponding schematic representation of compounds.

The degree of depolarization of *B. subtilis* WT168 cell membrane due to the effect mediated by the dendritic systems was measured by using a fluorescent voltage-sensitive dye Disc_3_(5) [32]. First, all the samples and the dye were initially measured without microorganisms (see Supplementary Material Figures S26 and S27) to rule out any interactions between the dye and the samples. Only in the case of bow-ties **11** and **15** dendrimers, a mild interaction with the dye was detected. Subsequently, the same measurements were carried out with the dendritic systems and the dye in the presence of bacteria (Figure 3 for **I–VII, 11, 15**/Disc_3_(5) and Figure 4 for **1–7**/Disc_3_(5)), using gramicidin as a positive control and sterile distilled water and DMSO 1% as a negative control.

The cationic imidazolium-containing dendritic systems **I–VII, 11**, and **15** led to a depolarization of the cell membrane, in some cases even reaching higher values than gramicidin (Figure 3). The depolarization activity clearly correlated with the increasing number of charges (generation), **I** < **II** < **III**, **IV** < **V**, and **11** < **15**. Although some differences could be ascribed to the topology, for example, with compounds **I**, **VI**, and **11** containing methyl as *N*-substituent, this factor did not seem to be decisive at least for the first generation of dendritic systems with four to five positive charges. It is tempting to attribute this behavior to the lipophilic-hydrophilic balance, since this tendency was not observed with mesityl compounds (**IV** and **VII**).

In the case of the second-generation dendrimers with eight positive charges (see **II** and **15**), shape appears to be important. Nevertheless, the nature of the imidazolium substituent also plays an important role, since the mesityl derivatives show a stronger depolarizing activity. In this sense, the mesityl spherical dendrimer **IV,** with less charges (first generation) than the methyl derivatives **II** and **III** (second and third generation) showed similar activities. Comparing the dendrons **VI** and **VII**, which have the same number of charges, the mesityl derivative was again more depolarizing. It should be pointed out that in dendrons, the number of charges was higher due to the possible protonation of the amines in aqueous media.

The introduction of a metal in the imidazole dendrimeric structure eliminates the positive charge on the dendritic system, which resulted in a reduction of cell membrane depolarization. All the neutral analogues containing Ag(I)-NHC were much less active than gramicidin except compound **1** at longer times (see Figure 4). Overall, the activity increased as the generation decreased, e.g., as observed with the spherical dendrimers **1**–**3.** An opposite behavior with regard to the substituent at the imidazolium ring was observed (compounds **1** and **4**, **2** and **5**, **6**,and **7**), with the methyl systems being more depolarizing than the mesityl ones. As a general trend, an increase in lipophilicity, either by increasing the generation or by the nature of the substituent on imidazole, causes decreased depolarization of the cell membrane.

Overall, these data suggest that for cationic compounds, an increase in the charge facilitates the depolarization of the cell membrane, which is then prone to lead to pore formation. For the neutral analogues, the lipophilic-hydrophilic balance was the most important characteristic to determine the depolarizing activity.

### 3.7. Cell Membrane Depolarization and Pore Formation: Microscopy

**Figure 5:**
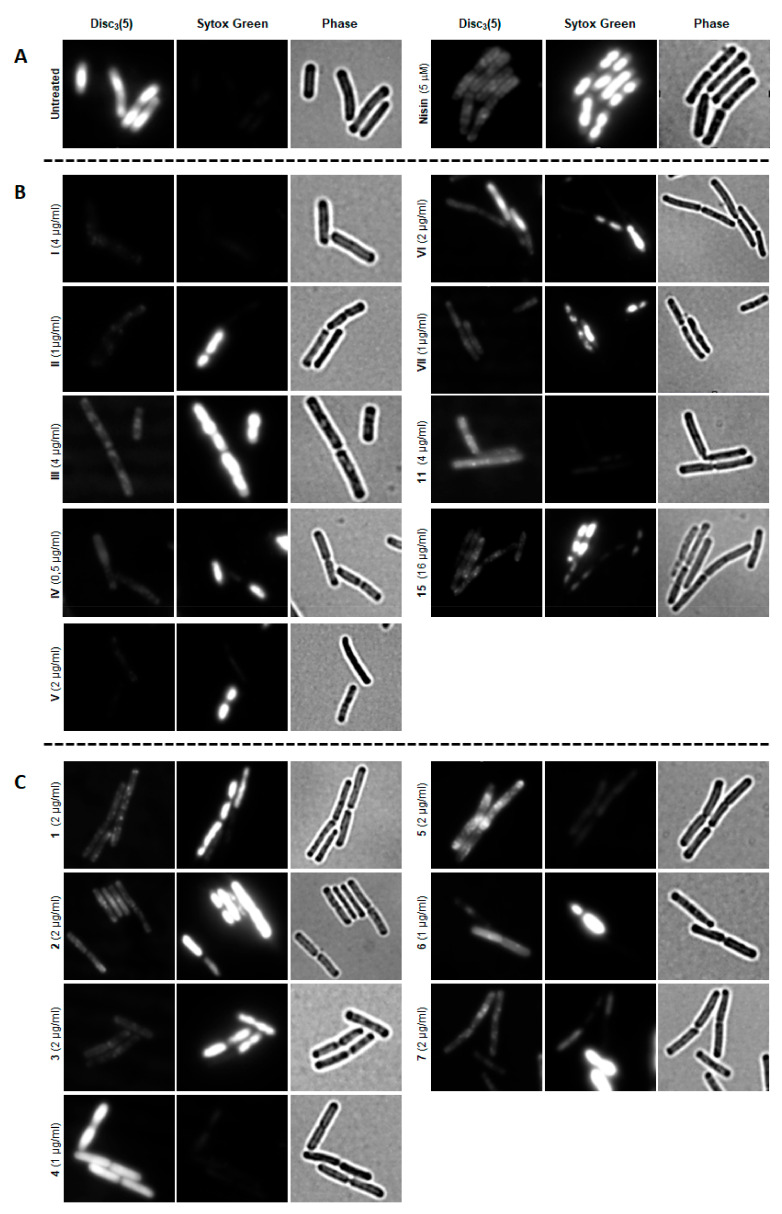
Selected microscopy images of cell membrane depolarization (Disc_3_(5)) and pore formation (Sytox Green) of *B. subtilis* WT168 in distilled sterile water (untreated) and in the presence of Nisin (**A**), in the presence of cationic dendritic systems (**I**–**VII**, **11**, and **15**) (**B**) and with their analogy containing Ag(I)-NHC (**1**–**7**) (**C**). Phase contrast images are also depicted. Incubation time 20 min.

Cell membrane depolarization and the subsequent pore formation of *B. subtillis* was also determined by microscopy, using the fluorescent voltage-sensitive Disc_3_(5) [32] and Sytox Green [47] stains, respectively. Low Disc_3_(5) emission hereby indicates a high degree of cell membrane depolarization, since the dye is released to the water medium. High emission values for Sytox Green dye, in contrast, means high capacity for pore formation, because it is a cell membrane-impermeable dye, which can only enter (and hence stain) permeabilized cells [48]. The selected concentration for each dendritic system was derived from the previous fluorescence assay. Selected microscopy images of cell membrane depolarization (Disc_3_(5)) and pore formation (Sytox Green) of *B. subtilis* WT168 in the presence of cationic imidazolium-containing dendritic systems (**I**–**VII**, **11**, and **15**) and their analogous containing Ag(I)-NHC units (**1**–**7**) were captured at 20 min post-treatment (Figure 5). Bacteria treated with sterile water were used as a negative control, where no depolarization and pore formation occurred (Figure 5A), that is, high values of Disc_3_(5) fluorescence but no Sytox signal were obtained. Nisin, a pore-forming peptide antibiotic, served as a positive control (Figure 5A). Here, high values of Sytox Green fluorescence were observed. DMSO 1% was not evaluated in this experiment, because no interference with the dyes was observed in the previous experiment. The results of the activity of the dendritic systems are shown in Figure 5B,C.

**Figure 6:**
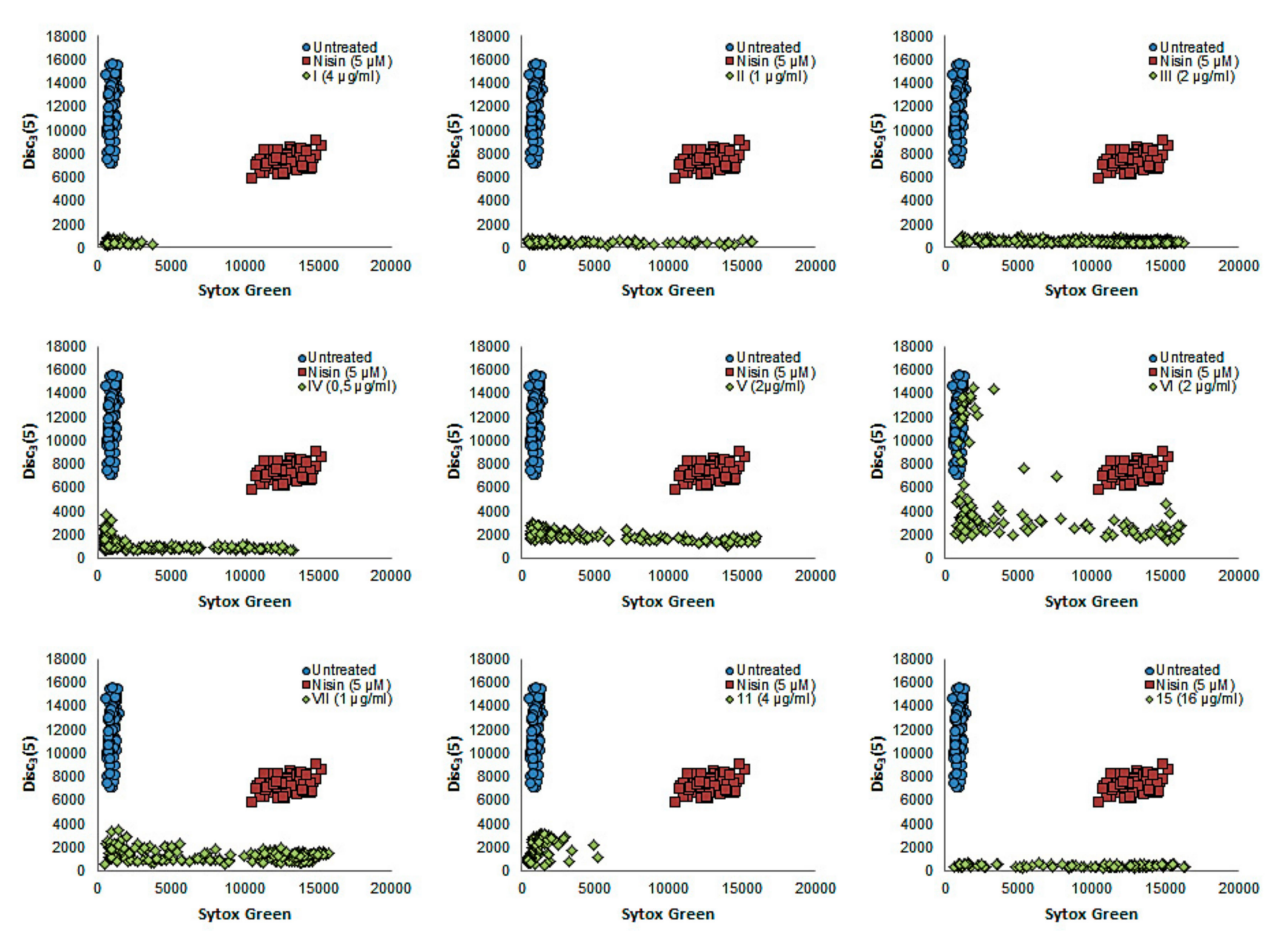
Depolarization and pore formation of *B. subtilis* WT168 induced by cationic imidazolium-containing dendritic systems. Bacteria treatment with: (blue) sterile distilled water; (red) Nissin as pore formation standard; (green) dendritic system.

**Figure 7:**
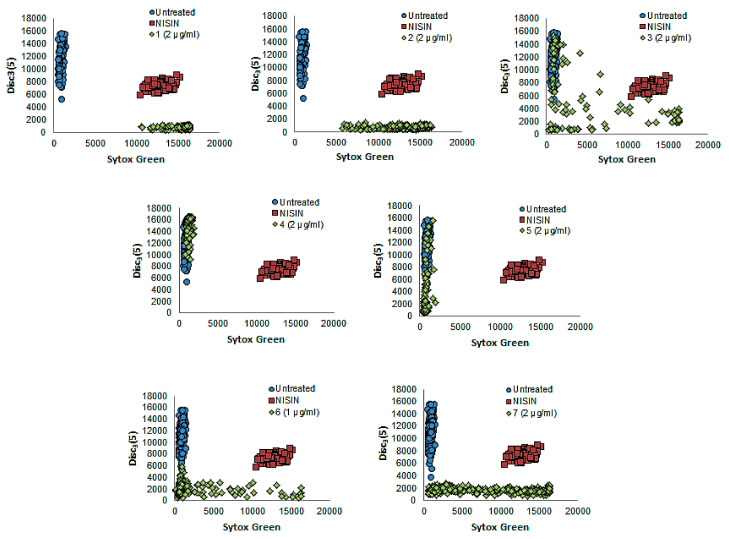
Depolarization and pore formation of *B. subtilis* WT168 induced by Ag(I)-NHC-containing dendritic systems. Bacteria treatment with: (blue) distillated sterile water; (red) Nissin as the pore formation standard; (green) dendritic system.

In order to gather statistically valid results on the tendency of imidazolium-containing dendrimers and Ag(I)-NHC dendrimers to act as depolarizing and pore-forming molecules, the data of approximately 200 bacterial cells were analyzed using the ImageJ-Fiji program (Figure 6; Figure 7). Bacteria treated only with distilled sterile water were depicted in blue as a negative control (no depolarization or pore formation), while nisin, depicted in red, served as a positive control for pore formation. The fluorescence values produced by the dendritic systems are shown in green (**I–VII**, **11**, **15** in Figure 6 and **1**–**7** in Figure 7).

As observed in Figure 5B and Figure 6, the imidazolium dendritic systems **I** and **11** could depolarize the cell membrane, but they were not able to form pores. However, the rest of the compounds was capable to induce both effects at different rates. The pore induction was clearly affected by the generation and enhanced by increasing the generation (see Figure 5B and Figure 6, compounds **I**–**III**, **IV**–**V,** and **11**, **15**). Dendrons **VI** and **VII**, with the same number of charges but different substituents on the imidazolium ring, presented a different behavior, and the mesityl derivative showed again higher activity than the methyl compound both in depolarization and pore formation. This effect could also be observed between compounds **I** and **IV**, demonstrating the influence of the substituent.

A different behavior was observed for the Ag(I)-NHC-containing dendrimers 1–5 (Figure 5C and Figure 7). In the case of dendrimers **1** and **2**, fast formation of pores occurred, while for the analogous metal-containing systems **4**–**5** having the mesityl substituent, no pore formation was observed, and cell membrane depolarization was only detected for the second-generation dendrimer **5**. This behavior must be ascribed to the non-existence of positive charges within these compounds, which notably reduced cell membrane depolarization, an effect that was modulated by the increased lipophilicity of the derivatives containing mesityl as a substituent on the imidazolium group. For dendrons **6** and **7**, the behavior was comparable to that observed for most of the imidazolium-containing compounds, suggesting that the terminal amine-protonated groups played the major role. The behavior described for the two types of dendritic molecule families by microscopy were in agreement with the results of cell membrane depolarization shown by the fluorescence assays, as expected.

### 3.8. Fatty Acids in Cell Membrane: Nile Red Assay by Microscopy

**Figure 8:**
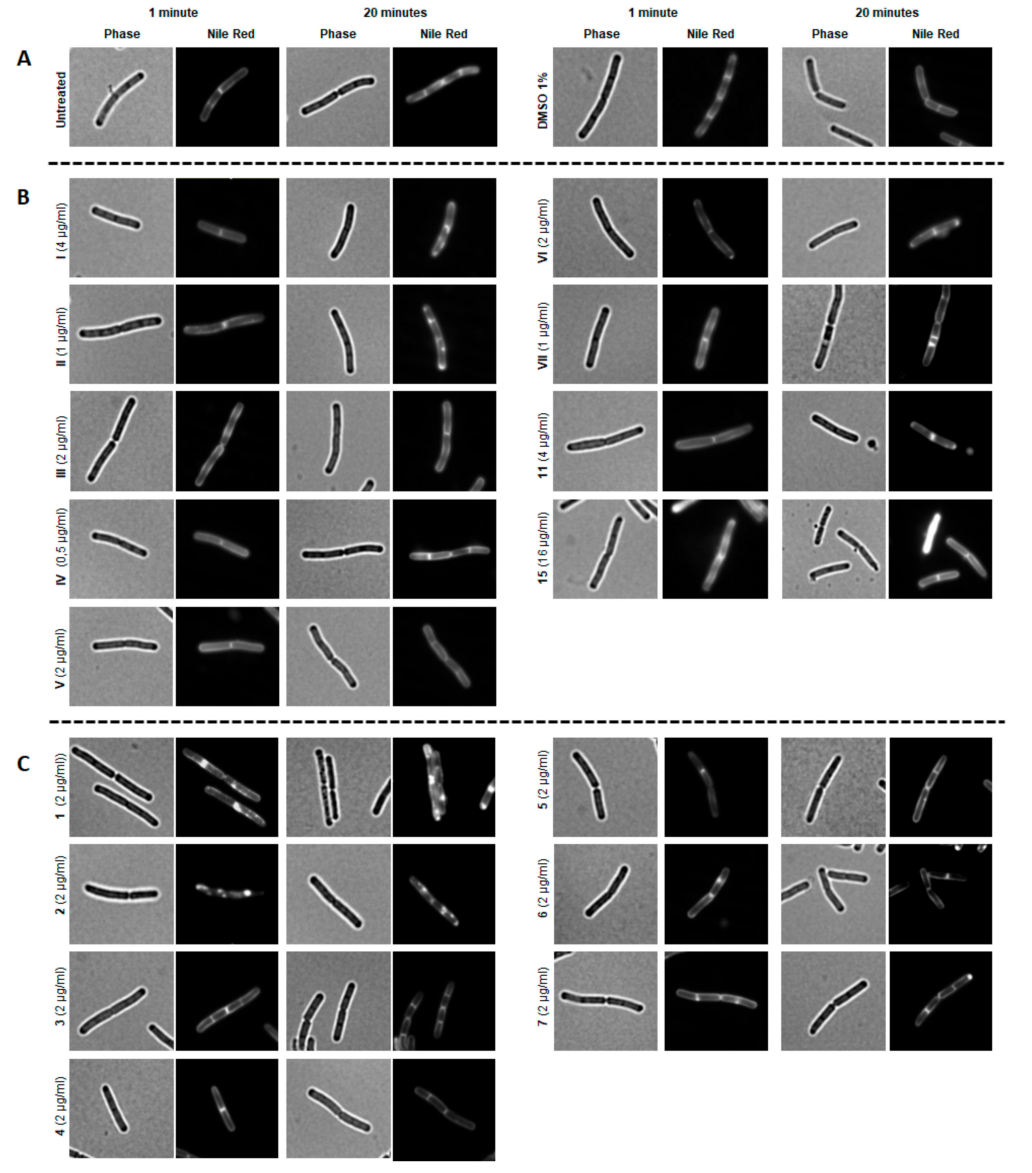
Microscopy images after 1 and 20 min of treatment with cationic (**B**) and Ag(I)-NHC-Ag (**C**) dendritic systems. Untreated and DMSO 1% are also depicted to control the proper growth of bacteria (**A**).

The change of the lipid distribution in the cell membrane [49] of *B. subtilis* as a result of the dendritic systems was determined by using Nile red. This uncharged fluorescent cell membrane dye incorporates into the lipid cell membrane based on its intrinsic hydrophobicity, enabling a sensitive readout for physical changes of lipid environments [50,51,52]. *B. subtilis* was grown and treated with each sample using concentrations just below the MIC values. After staining with Nile red and placing the bacteria already treated with each sample in agarose pads, fluorescence was measured after 1 and 20 min of treatment. Figure 8A shows the normal pattern of Nile red distribution in the cell membrane of untreated bacteria. The same distribution was observed after treatment with DMSO 1% (Figure 8A). After one minute of treatment, there were no noticeable differences in Nile red distribution. In contrast, after 20 min, dendrimers containing methylimidazolium salts in the periphery (**I**–**II**) led to the formation of lipid domains, indicating that they were able to interact with the cell membrane and perturb the distribution of fatty acids (Figure 8B). Their analogy with Ag(I)-NHC (**1**–**2**) generates the same domains much faster, that is, already after one minute of treatment (see Figure 8C). Hence, the organometallic fragment significantly enhanced their activity, probably due to their lower polarity, although the release of the silver ions from the complex can also not be ruled out.

Again, AgNO_3_ alone did not result in lipid domain formation, emphasizing the importance of the organometallic fragment and the dendritic structure (see Supplementary Material Figure S24D). Therefore, although the changes observed in the distribution of fatty acids depended on different factors like topology or the imidazolium substituent, the presence of Ag(I)-NHC can be considered the main difference significantly accelerating the process of cell membrane remodeling.

### 3.9. Protein Delocalization in the Cell Membrane: MreB and MinD

**Figure 9:**
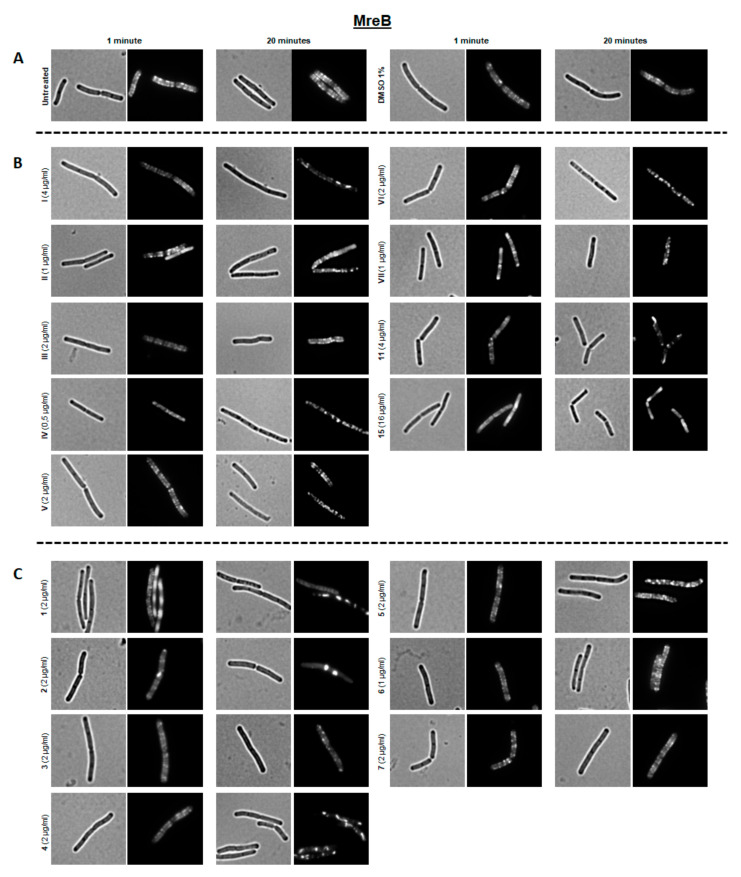
Representative microscopy images of MreB protein delocalization: (**A**) with untreated bacteria and with addition of DMSO 1% to control the proper growth of bacteria, (**B**) in the presence of cationic dendritic systems, (**C**) and in the presence of Ag(I)-NHC dendritic systems.

Considering that the dendritic systems affect the bacterial envelope at the level of the cell membrane, their effect on the localization of two crucial cell membrane-associated proteins involved in cell wall synthesis and cell shape (MreB) and cell division regulation (MinD) [53] were next tested by challenging two *B. subtilis* reporter strains expressingGFP-tagged versions of each protein. After incubation with the corresponding compounds for 1 and 20 min, fluorescence pictures were obtained and probed for delocalization of MreB or MinD, respectively. Figure 9A and Figure S28A (see Supplementary Material) show the normal protein distribution patterns of MreB and MinD, respectively, indicating the proper functioning of bacteria and that the addition of DMSO did not affect the bacteria. In general, a common delocalization of MinD (see Supplementary Material, Figure S28) in the presence of the compounds was observed already after one minute of treatment. It is important to point out the strong effect observed for MreB when treated with **1** and **2** (Figure 9C), leading to a clear formation of protein patches after one minute of treatment. In contrast, for the analogous cationic compounds **I** and **II**, the same effect was only produced after 20 min. This tendency corroborated the previous behavior observed with Nile red that the presence of Ag(I)-NHC fragments significantly enhanced the disorganization of the cell membrane, and hence also associated proteins. Again, the use of AgNO_3_ alone did not alter MinD or MreB localization significantly, with only a slight protein dissipation after 20 min (see Supplementary Material Figure S24D).

## 4. Conclusions

Two new families of dendritic systems containing Ag(I)-NHC and metal-free bow-tie systems were successfully synthesized. Their antibacterial behavior was comprehensively studied and the differences in activity seem to be a compromise between different parameters like shape, number of positive charges, generation, hydrophobicity, or the nature of the imidazolium substituent, i.e., the lipophilic-hydrophilic balance in the molecule is decisive for their antibacterial activity.

However, differences in the mode of action between cationic imidazolium species and those containing silver atoms were observed and focused on the disruption of the cell membrane. While dendritic systems with Ag(I) were able to induce two biosensors of cell envelope stress, no significant stimuli were generated by the cationic imidazolium derivatives, indicating that only the silver dendritic systems can attack this particular cellular target. The consistently negative results of AgNO_3_ alone demonstrates that the organometallic fragment is necessary for envelope perturbations.

Subsequent comprehensive studies on cell membrane depolarization and pore formation demonstrated that cationic systems strongly depolarize the cell membrane, while in the silver(I) metallodendrimers, this property was notably reduced, as a consequence of modifying the charge by obtaining neutral compounds. The studies of modification of the lipid distribution in the cell membrane or protein delocalization with fluorescent proteins showed a surprisingly fast perturbation after one minute of treatment only with the silver metallodendrimers, while slower perturbation was observed for the cationic imidazolium dendrimers.

In light of these modes of action, it is noteworthy to highlight important differences between silver (I) metallodendrimers and the inorganic salt AgNO_3_. In contrast to the first, the latter neither biosensor stimulation nor fatty acids or proteins from the cell membrane modifications were observed, emphasizing the importance of the organometallic fragment in the structure as well as the polyvalency of dendrimers as decisive features in the internalization of the metal for their antibacterial action.

This comparative antibacterial study opens the door to the use of cationic imidazolium dendrimers or Ag(I)-NHC metallodendrimers as promising candidates for their use as antibacterial pharmaceutics based on the polyvalence concept where both the peripheral groups and the dendritic skeleton may play important roles.

## Figures and Tables

**Table 1:** Bacterial strains used for different microbiological assays.

Bacterial Strain	Description	Source/Reference
	MIC and MBC assays	
*Escherichia coli*	CECT515, Gram-negative	Spanish Type Culture Collection (CECT)
*Staphylococcus aureus*	CECT 2404, Gram-positive	Spanish Type Culture Collection (CECT)
*Bacillus subtilis*	Wild-typ, trpC2	lab strain
	Biosensors assay	
*B. subtilis TMB2009*	W168 *sacA*::pJH*lux*104 (P*_psdA_*-*lux*)	[29]
*B. subtilis TMB2299*	W168 *sacA*::pASp3C*lux*01 (P*_pspA_*-*lux*)	[29]
*B. subtilis TMB3417*	W168 *sacA*::pM133C*lux*03 (P*_bceA_*-*lux*)	lab collection
*B. subtilis TMB3561*	W168 *sacA*::pBS3C*lux*01 (P*_yxdL_*-*lux*)	[30]
*B. subtilis TMB3791*	W168 *sacA*::pBS3C*lux*-P*_fabHB_* (P*_fabHB_*-*lux*)	lab collection
*B. subtilis TMB3822*	W168 *sacA*::pBS3C*lux*-P*_liaI_* (P*_liaI_*-*lux*)	[31]
Fluorescence assay (Microscopy) and Nile red		
*Bacilus subtilis*	Wild-typ, trpC2	lab strain
Protein delocalization assay		
*Bacilus subtilis*	KS69 *amyE*::*spc* P*_xylA_*-*msfgfp*-*mreB*	[32]
*Bacilus subtilis*	KS64 *amyE*::*spec* P*_xylA_*-*gfp*-*minD*	[33]

**Table 2:** MIC and MBC (µg/mL) values of the cationic dendrimers (**I**–**V**), dendrons (**VI**–**VII**) and bow-ties (**11**, **15**) containing imidazolium salts.

Compound ^a^	*Escherichia coli*		*Staphylococcus aureus*		*Bacillus subtilis*	
	*MIC*	*MBC*	*MIC*	*MBC*	*MIC*	*MBC*
**I (4)**	32	32	8	16	2	2
**II (8)**	4	4	1	4	0.5	1
**III (16)**	8	16	4	4	2	2
**IV (4)**	1	1	0.5	0.5	0.5	1
**V (8)**	16	16	2	2	4	4
**VI (1 + 4) ^b^**	4	4	8	8	1	1
**VII (1 + 4) ^b^**	4	4	2	2	1	1
**11 (4)**	4	4	1	2	2	4
**15 (8)**	128	128	16	16	8	8

^a^ In brackets, number of positive charges. ^b^ Dendrons **VI** and **VII** contain two different types of charges: four positives charges from the peripheral amines and one positive charge from the imidazolium ring.

**Table 3:** MIC and MBC (µg/mL) values of the dendrimers (**1**–**5**) and dendrons (**6**–**7**) containing Ag(I)-NHC.

Compound ^a^	*Escherichia coli*		*Staphylococcus aureus*		*Bacillus subtilis*	
	*MIC*	*MBC*	*MIC*	*MBC*	*MIC*	*MBC*
**1 (4)**	4	4	4	4	1	2
**2 (8)**	4	4	2	2	1	2
**3 (16)**	8	8	4	4	2	4
**4 (4)**	2	2	1	2	1	2
**5 (8)**	8	8	4	4	4	4
**6 (1)**	4	4	2	2	1	1
**7 (1)**	2	2	2	2	1	2
**AgNO_3_**	-	-	-	-	0.25	0.5

^a^ In brackets, number of silver atoms.

## Supplementary Materials

The following are available online at https://www.mdpi.com/1999-4923/12/10/968/s1, Figure S1. Schematic representations of G_n_Si(RImidAgCl)_m_ compounds 1-5; Figure S2. Schematic representations of (RImidAgCl)G_2_(S(CH_2_)_2_NMe_2_)_4_ compounds 6-7; Figure S3. ^1^H-NMR and ^13^C{^1^H}-NMR of G_1_Si(CH_2_MeImidAgCl)_4_ (1) in CDCl_3_; Figure S4. ^1^H-NMR and ^13^C{^1^H}-NMR of G_1_Si(CH_2_MesImidAgCl)_4_ (4) in CDCl_3_; Figure S5. ^1^H-NMR and ^13^C{^1^H}-NMR of AgClMeImidG_2_(S(CH_2_)_2_NMe_2_)_4_ (6) in CDCl_3_; Figure S6. ^1^H-NMR, ^13^C{^1^H}-NMR and HSQC of AgClMesImidG_2_(S(CH_2_)_2_NMe_2_)_4_ (7) in CDCl_3;_ Figure S7. 1H-NMR study of G1Si(CH2MeImidAgCl)4 (1) in D2O:DMSO-d6 (1:1) at different times; Figure S8. ^1^H-NMR study of G_2_Si(CH_2_MeImidAgCl)_8_ (2) in D_2_O:DMSO-d^6^ (1:1) at different times; Figure S9. 1H-NMR study of G3Si(CH2MeImidAgCl)16 (3) in D2O:DMSO-d6 (1:1) at different times; Figure S10. ^1^H-NMR study of G_1_Si(CH_2_MesImidAgCl)_4_ (4) in D_2_O:DMSO-d^6^ (1:1) at different times; Figure S11. ^1^H-NMR study of AgClMeImidG_2_(S(CH_2_)_2_NMe_2_)_4_ (6) in D_2_O:DMSO-d^6^ (1:1) at different times; Figure S12. ^1^H-NMR study of AgClMesImidG_2_(S(CH_2_)_2_NMe_2_)_4_ (7) in D_2_O(WSAT):DMSO-d^6^ (1:1) at different times; Figure S13. Schematic representations of (BrMeImid(CH_2_)_4_Si)_m_G_n_[OC_6_H_4_O]G_n_(Si(CH_2_)_4_ImidMeBr)_m_ Compounds 11-15; Figure S14. ^1^H-NMR of (Cl(CH_3_)_2_Si)_2_G_1_[OC_6_H_4_O]G_1_(Si(CH_3_)_2_Cl)_2_ (8) in CDCl_3_; Figure S15. ^1^H-NMR of (H(CH_3_)_2_Si)_2_G_1_[OC_6_H_4_O]G_1_(Si(CH_3_)_2_H)_2_ (9) in CDCl_3_; Figure S16. ^1^H-NMR of (Br(CH_2_)_4_Si)_2_G_1_[OC_6_H_4_O]G_1_(Si(CH_2_)_4_Br)_2_ (10) in CDCl_3_; Figure S17. ^1^H-NMR and ^13^C{^1^H}-NMR of (BrMeImid(CH_2_)_4_Si)_2_G_1_[OC_6_H_4_O]G_1_(Si(CH_2_)_4_ImidMeBr)_2_ (11) in CD_3_OD; Figure S18. ESI-TOF [M-Br]^+^ (BrMeImid(CH_2_)_4_Si)_2_G_1_[OC_6_H_4_O]G_1_(Si(CH_2_)_4_ImidMeBr)_2_ (11); Figure S19. ^1^H-NMR of (Cl(CH_3_)_2_Si)_4_G_2_[OC_6_H_4_O]G_2_(Si(CH_3_)_2_Cl)_4_ (12) in CDCl_3_; Figure S20. ^1^H-NMR of (H(CH_3_)_2_Si)_4_G_2_[OC_6_H_4_O]G_2_(Si(CH_3_)_2_H)_4_ (13) in CDCl_3_; Figure S21. ^1^H-NMR of (Br(CH_2_)_4_Si)_4_G_2_[OC_6_H_4_O]G_2_(Si(CH_2_)_4_Br)_4_ (14) in CDCl_3_; Figure S22. ^1^H-NMR, ^13^C{^1^H}-NMR and HSQC of (BrMeImidButSi)_4_G_2_[OC_6_H_4_O]G_2_(SiButImidMeBr)_4_ (15) in CD_3_OD; Figure S23. Biosensors induction in presence of dendrimers 1-5 and dendrons 6-7 containing Ag(I)- NHC carbenes in the periphery; Figure S24. Results in presence of AgNO_3_: (A) Biosensor induction assay; (B) Fluorescence assay, control graph (left), depolarization graph (right); (C) Fluorescence assay. Microscopy; (D) NileRed and protein delocalization; Figure S25. Biosensors induction in presence of dendrimers I-V, dendrons VI-VII and bow-ties 11, 15 containing imidazolium salts; Figure S26. Disc_3_(5) fluorescence control graphs of cell membrane depolarization assay of *B. subtilis* WT168 in presence of compounds I-VII, 11 and 15; Figure S27. Disc_3_(5) fluorescence control graphs of cell membrane depolarization assay of *B. subtilis* WT168 in presence of compounds 1-7; Figure S28. Representative microscopy images of MinD protein delocalization in presence of cationic (B) and Ag(I)-NHC (C) dendritic systems. Untreated and DMSO 1% are also depicted, to control the proper growth of bacteria (A).; Table S1. Comparative table detailing the activity of AgNO_3_ with the silver dendrimers 1-7 in molar ratio or *per* silver atom on *B. subtilis*.

## References

[1] García-Gallego S., Franci G., Falanga A., Gómez R., Folliero V., Galdiero S., De La Mata F.J., Galdiero S. (2017). Function Oriented Molecular Design: Dendrimers as Novel Antimicrobials. Molecules.

[2] Wan J., Alewood P.F. (2016). Peptide-Decorated Dendrimers and Their Bioapplications. Angew. Chem. Int. Ed..

[3] Venkataraman S., Lee A.L.Z., Tan J.P.K., Ng Y.C., Lin A.L.Y., Yong J.Y.K., Yi G., Zhang Y., Lim I.J., Phan T.T. (2019). Functional cationic derivatives of starch as antimicrobial agents. Polym. Chem..

[4] Zhou C., Wang F., Chen H., Li M., Qiao F., Liu Z., Hou Y., Wu C., Fan Y., Liu L. (2016). Selective Antimicrobial Activities and Action Mechanism of Micelles Self-Assembled by Cationic Oligomeric Surfactants. ACS Appl. Mater. Interfaces.

[5] Riduan S.N., Zhang Y. (2013). Imidazolium salts and their polymeric materials for biological applications. Chem. Soc. Rev..

[6] Hryniewicka A., Malinowska M., Hauschild T., Pieczul K., Morzycki J.W. (2019). Synthesis and antimicrobial properties of steroid-based imidazolium salts. J. Steroid Biochem. Mol. Biol..

[7] Sharma D., Narasimhan B., Kumar P., Judge V., Narang R., De Clercq E., Balzarini J. (2009). Synthesis, antimicrobial and antiviral evaluation of substituted imidazole derivatives. Eur. J. Med. Chem..

[8] Jin Z. (2011). Muscarine, imidazole, oxazole, and thiazole alkaloids. Nat. Prod. Rep..

[9] Hindi K.M., Panzner M.J., Tessier C.A., Cannon C.L., Youngs W.J. (2009). The Medicinal Applications of Imidazolium Carbene−Metal Complexes. Chem. Rev..

[10] Liu W., Gust R. (2016). Update on metal N-heterocyclic carbene complexes as potential anti-tumor metallodrugs. Co-ord. Chem. Rev..

[11] Marion N., Díez-González S., Nolan S.P. (2007). N-heterocyclic carbenes as organocatalysts. Angew. Chem. Int. Ed..

[12] Biffis A., Cipani M., Tubaro C., Basato M., Costante M., Bressan E., Venzo A., Graiff C. (2013). Dinuclear complexes of silver(i) and gold(i) with macrocyclic dicarbene ligands bearing a 2,6-lutidinyl bridge: Synthesis, structural analysis and dynamic behaviour in solution. New J. Chem..

[13] Hopkinson M.N., Richter C., Schedler M., Glorius F. (2014). An overview of N-heterocyclic carbenes. Nat. Cell Biol..

[14] Liang X., Luan S., Yin Z.-Q., He M., He C., Yin L., Zou Y., Yuan Z., Li L., Song X. (2018). Recent advances in the medical use of silver complex. Eur. J. Med. Chem..

[15] Liu W., Gust R. (2013). Metal N-heterocyclic carbene complexes as potential antitumor metallodrugs. Chem. Soc. Rev..

[16] Fuentes-Paniagua E., Hernández-Ros J.M., Sánchez-Milla M., Camero M.A., Maly M., Pérez-Serrano J., Copa-Patiño J.L., Sánchez-Nieves J., Soliveri J., Gómez R. (2014). Carbosilane cationic dendrimers synthesized by thiol–ene click chemistry and their use as antibacterial agents. RSC Adv..

[17] Muñoz-Bonilla A., Fernández-García M. (2012). Polymeric materials with antimicrobial activity. Prog. Polym. Sci..

[18] Svenson S., Tomalia D.A. (2012). Dendrimers in biomedical applications—Reflections on the field. Adv. Drug Deliv. Rev..

[19] Nanjwade B.K., Bechra H.M., Derkar G.K., Manvi F., Nanjwade V.K. (2009). Dendrimers: Emerging polymers for drug-delivery systems. Eur. J. Pharm. Sci..

[20] Gitsov I., Lin C. (2005). Dendrimers—Nanoparticles with Precisely Engineered Surfaces. Curr. Org. Chem..

[21] Gutierrez-Ulloa C.E., Sepúlveda-Crespo D., García-Broncano P., Malý M., Muñoz-Fernández M.A., De La Mata F.J., Gómez R. (2019). Synthesis of bow-tie carbosilane dendrimers and their HIV antiviral capacity: A comparison of the dendritic topology on the biological process. Eur. Polym. J..

[22] Sandoval-Yañez C., Rodriguez C.C. (2020). Dendrimers: Amazing Platforms for Bioactive Molecule Delivery Systems. Materials.

[23] Gladitz M., Reinemann S., Radusch H.-J. (2009). Preparation of Silver Nanoparticle Dispersions via a Dendritic-Polymer Template Approach and their Use for Antibacterial Surface Treatment. Macromol. Mater. Eng..

[24] Balogh L.P., Swanson D.R., Tomalia N.A., Hagnauer G.L., McManus A.T. (2001). Dendrimer−Silver Complexes and Nanocomposites as Antimicrobial Agents. Nano Lett..

[25] Krasheninina O.A., Apartsin E.K., Fuentes E., Szulc A., Ionov M., Venyaminova A.G., Shcharbin D., De La Mata F.J., Bryszewska M., Gόmez R. (2019). Complexes of Pro-Apoptotic siRNAs and Carbosilane Dendrimers: Formation and Effect on Cancer Cells. Pharmaceutics.

[26] Sánchez-Milla M., Muñoz-Moreno L., Sánchez-Nieves J., Malý M., Gómez R., Carmena M.J., De La Mata F.J. (2019). Anticancer Activity of Dendriplexes against Advanced Prostate Cancer from Protumoral Peptides and Cationic Carbosilane Dendrimers. Biomacromolecules.

[27] Wolf D., Mascher T. (2016). The applied side of antimicrobial peptide-inducible promoters from Firmicutes bacteria: Expression systems and whole-cell biosensors. Appl. Microbiol. Biotechnol..

[28] Rodríguez-Prieto T., Fattori A., Camejo C., De La Mata F.J., Cano J., Ottaviani M.F., Gómez R. (2020). Synthesis of imidazolium-terminated carbosilane dendrimers and dendrons and study of their interactions with a cell membrane model. Eur. Polym. J..

[29] Höfler C., Heckmann J., Fritsch A., Popp P., Gebhard S., Fritz G., Mascher T. (2016). Cannibalism stress response in Bacillus subtilis. Microbiology.

[30] Müller A., Wolf D., Gutzeit H.O. (2017). The black soldier fly, Hermetia illucens—A promising source for sustainable production of proteins, lipids and bioactive substances. Zeitschrift für Naturforschung C.

[31] Popp P.F., Dotzler M., Radeck J., Bartels J., Mascher T. (2017). The Bacillus BioBrick Box 2.0: Expanding the genetic toolbox for the standardized work with Bacillus subtilis. Sci. Rep..

[32] Scheinpflug K., Wenzel M., Krylova O., Bandow J.E., Dathe M., Strahl H. (2017). Antimicrobial peptide cWFW kills by combining lipid phase separation with autolysis. Sci. Rep..

[33] Winkel J.D.T., Gray D.A., Seistrup K.H., Hamoen L.W., Strahl H. (2016). Analysis of Antimicrobial-Triggered Membrane Depolarization Using Voltage Sensitive Dyes. Front. Cell Dev. Biol..

[34] ISO 20776-1: 2006 Clinical Laboratory Testing and in Vitro Diagnostic Test Systems–Susceptibility Testing of Infectious Agents and Evaluation of Performance of Antimicrobial Susceptibility Test Devices–Part 1: Reference Method for Testing the in Vitro Activity of Antimicrobial Agents against Rapidly Growing Aerobic Bacteria Involved in Infectious Diseases. https://www.iso.org/standard/41630.html.

[35] Popp P.F., Benjdia A., Strahl H., Berteau O., Mascher T. (2020). The Epipeptide YydF Intrinsically Triggers the Cell Envelope Stress Response of Bacillus subtilis and Causes Severe Membrane Perturbations. Front. Microbiol..

[36] Revilla-Guarinos A., Dürr F., Popp P.F., Döring M., Mascher T. (2020). Amphotericin B Specifically Induces the Two-Component System LnrJK: Development of a Novel Whole-Cell Biosensor for the Detection of Amphotericin-Like Polyenes. Front. Microbiol..

[37] Kepplinger B., Morton-Laing S., Seistrup K.H., Marrs E.C.L., Hopkins A., Perry J.D., Strahl H., Hall M.J., Errington J., Allenby N. (2017). Mode of Action and Heterologous Expression of the Natural Product Antibiotic Vancoresmycin. ACS Chem. Biol..

[38] Schindelin J., Arganda-Carreras I., Frise E., Kaynig V., Longair M., Pietzsch T., Preibisch S., Rueden C., Saalfeld S., Schmid B. (2012). Fiji: An open-source platform for biological-image analysis. Nat. Methods.

[39] Ducret A., Quardokus E.M., Brun Y.V. (2016). MicrobeJ, a tool for high throughput bacterial cell detection and quantitative analysis. Nat. Microbiol..

[40] Bertrand B., Bodio E., Richard P., Picquet M., Le Gendre P., Casini A. (2015). Gold(I) N-heterocyclic carbene complexes with an “activable” ester moiety: Possible biological applications. J. Organomet. Chem..

[41] Li D., Ollevier T. (2020). Mechanism studies of oxidation and hydrolysis of Cu(I)–NHC and Ag–NHC in solution under air. J. Organomet. Chem..

[42] Hindi K.M., Siciliano T.J., Durmus S., Panzner M.J., Medvetz D.A., Reddy D.V., Hogue L.A., Hovis C.E., Hilliard J.K., Mallet R.J. (2008). Synthesis, Stability, and Antimicrobial Studies of Electronically Tuned Silver AcetateN-Heterocyclic Carbenes. J. Med. Chem..

[43] Seyferth D., Son D.Y., Rheingold A.L., Ostrander R.L. (1994). Synthesis of an Organosilicon Dendrimer Containing 324 Si-H Bonds. Organometallics.

[44] Johnson N.A., Southerland M.R., Youngs W.J. (2017). Recent Developments in the Medicinal Applications of Silver-NHC Complexes and Imidazolium Salts. Molecules.

[45] Kobras C.M., Mascher T., Gebhard S. (2016). Application of a Bacillus subtilis Whole-Cell Biosensor (PliaI-lux) for the Identification of Cell Wall Active Antibacterial Compounds. Methods Mol. Biol..

[46] Staroń A., Finkeisen D.E., Mascher T. (2010). Peptide Antibiotic Sensing and Detoxification Modules ofBacillus subtilis. Antimicrob. Agents Chemother..

[47] Alkhatib Z., Lagedroste M., Fey I., Kleinschrodt D., Abts A., Smits S.H.J. (2014). Lantibiotic Immunity: Inhibition of Nisin Mediated Pore Formation by NisI. PLoS ONE.

[48] Lamsa A., Liu W.-T., Dorrestein P.C., Pogliano K. (2012). The Bacillus subtilis cannibalism toxin SDP collapses the proton motive force and induces autolysis. Mol. Microbiol..

[49] Strahl H., Bürmann F., Hamoen L.W. (2014). The actin homologue MreB organizes the bacterial cell membrane. Nat. Commun..

[50] Greenspan P., Fowler S.D. (1985). Spectrofluorometric studies of the lipid probe, nile red. J. Lipid Res..

[51] Kucherak O.A., Oncul S., Darwich Z., Yushchenko D.A., Arntz Y., Didier P., Mély Y., Klymchenko A.S. (2010). Switchable Nile Red-Based Probe for Cholesterol and Lipid Order at the Outer Leaflet of Biomembranes. J. Am. Chem. Soc..

[52] Mukherjee S., Raghuraman H., Chattopadhyay A. (2007). Membrane localization and dynamics of Nile Red: Effect of cholesterol. Biochim. Biophys. Acta Biomembr..

[53] Müller A., Wenzel M., Strahl H., Grein F., Saaki T.N.V., Kohl B., Siersma T., Bandow J.E., Sahl H.-G., Schneider T. (2016). Daptomycin inhibits cell envelope synthesis by interfering with fluid membrane microdomains. Proc. Natl. Acad. Sci. USA.

