# Supplementary material for: Silver (I) N-Heterocyclic Carbenes Carbosilane Dendritic Systems and Their Imidazolium-Terminated Analogues as Antibacterial Agents: Study of Their Mode of Action
Source: Pharmaceutics. 2020 Oct 14;12(10):968. doi: 10.3390/pharmaceutics12100968 (PMC7650833; doi:10.3390/pharmaceutics12100968)
Supplement: Supplementary file 1 [file pharmaceutics-12-00968-s001.zip › pharmaceutics-946285-supplementary.docx]

Supplementary Material: Silver (I) N-Heterocyclic Carbenes Carbosilane Dendritic Systems and Their Imidazolium-Terminated Analogues as Antibacterial Agents: Study of Their Mode of Action

Tamara Rodríguez-Prieto, Philipp F. Popp, José Luis Copa-Patiño, F. Javier de la Mata, Jesús Cano, Thorsten Mascher and Rafael Gómez


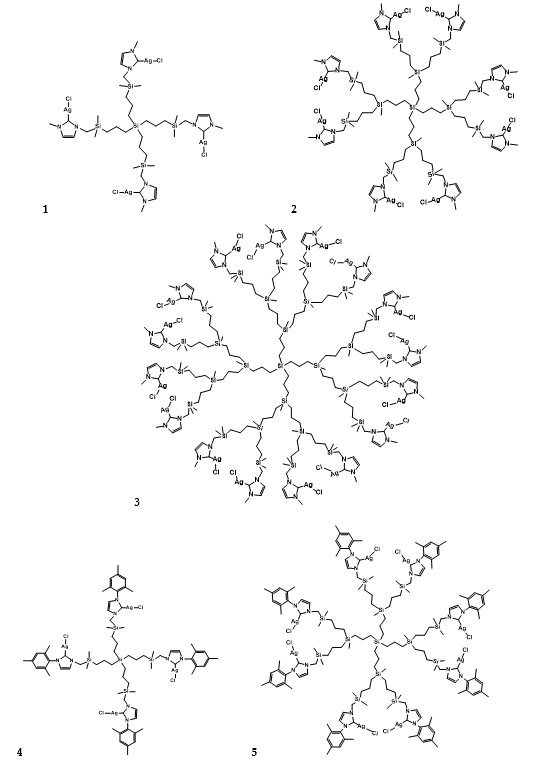


**Figure S1.** Schematic representations of G_n_Si(RImidAgCl)_m_ compounds 1-5.

 **7**

**Figure S2.** Schematic representations of (RImidAgCl)G_2_(S(CH_2_)_2_NMe_2_)_4_ compounds 6-7.


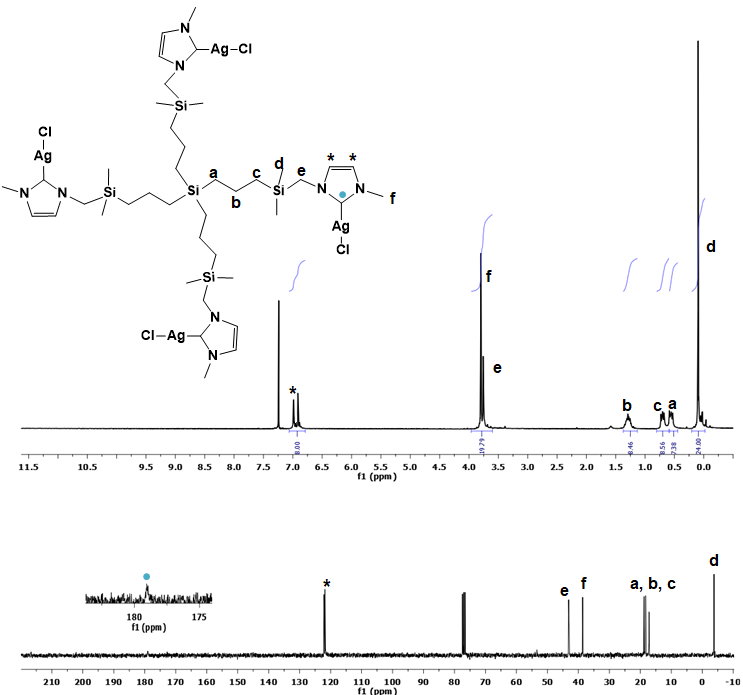


**Figure S3.** ^1^H-NMR and ^13^C{^1^H}-NMR of G_1_Si(CH_2_MeImidAgCl)_4_ (1) in CDCl_3_**_._**


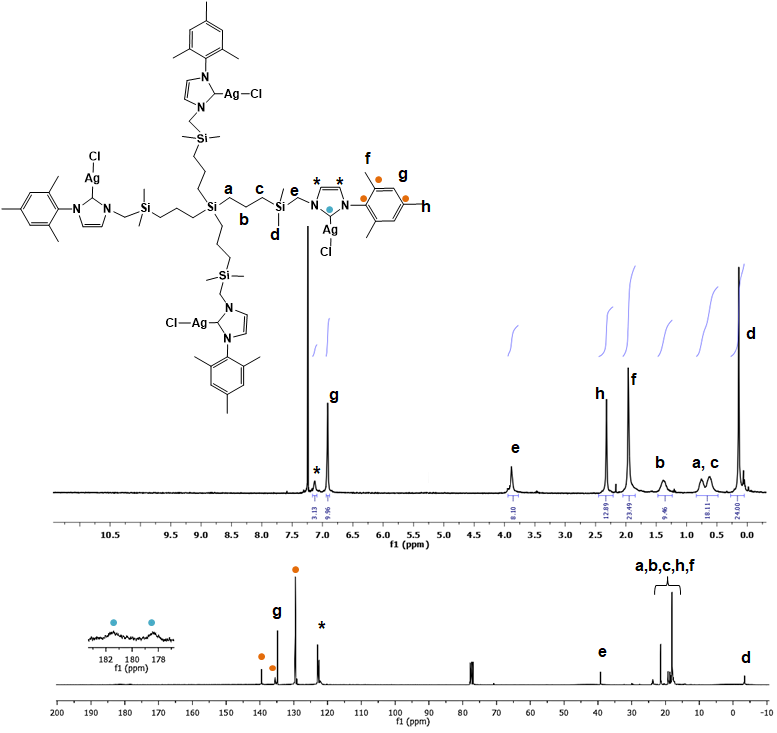


**Figure S4.** ^1^H-NMR and ^13^C{^1^H}-NMR of G_1_Si(CH_2_MesImidAgCl)_4_ (4) in CDCl_3_**_._**


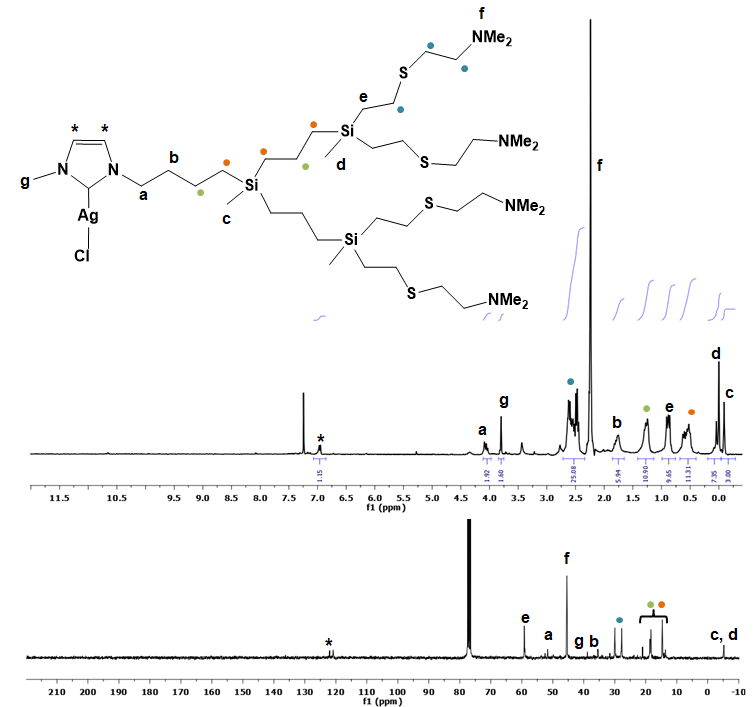


**Figure S5.** ^1^H-NMR and ^13^C{^1^H}-NMR of AgClMeImidG_2_(S(CH_2_)_2_NMe_2_)_4_ (6) in CDCl_3_**_._**


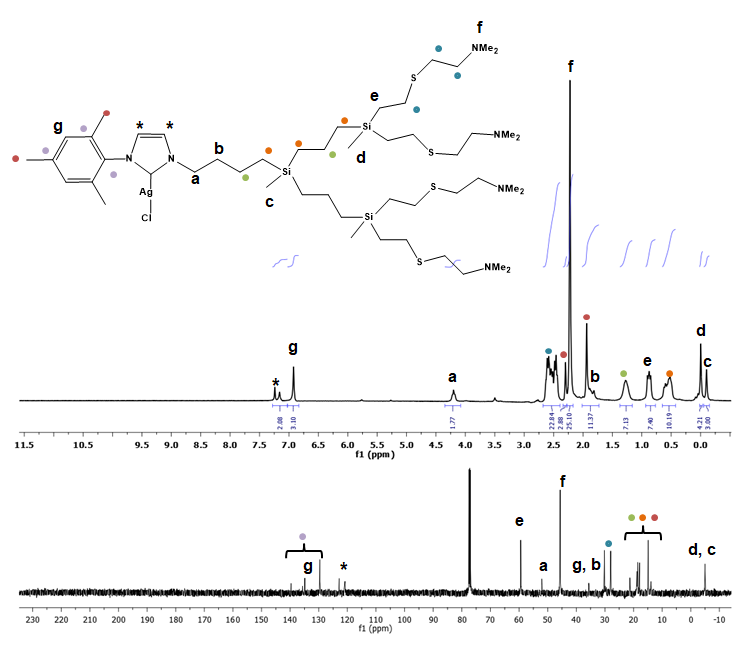

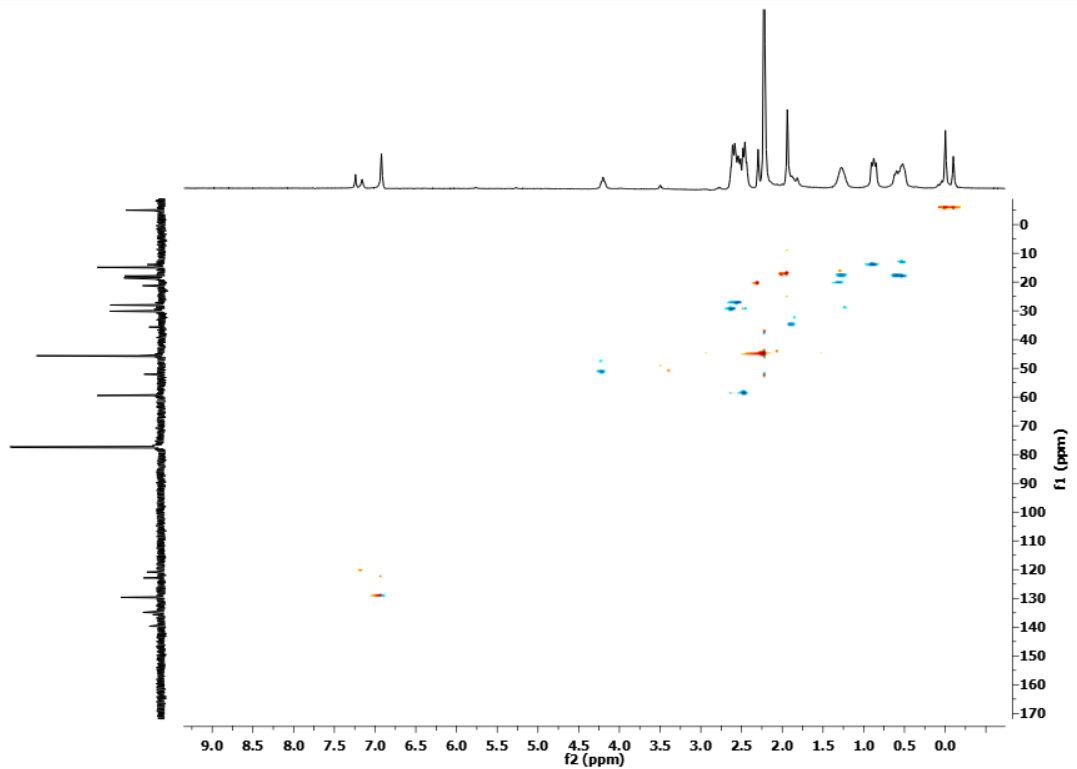


**Figure S6.** ^1^H-NMR, ^13^C{^1^H}-NMR and HSQC of AgClMesImidG_2_(S(CH_2_)_2_NMe_2_)_4_ (7) in CDCl_3_**_._**


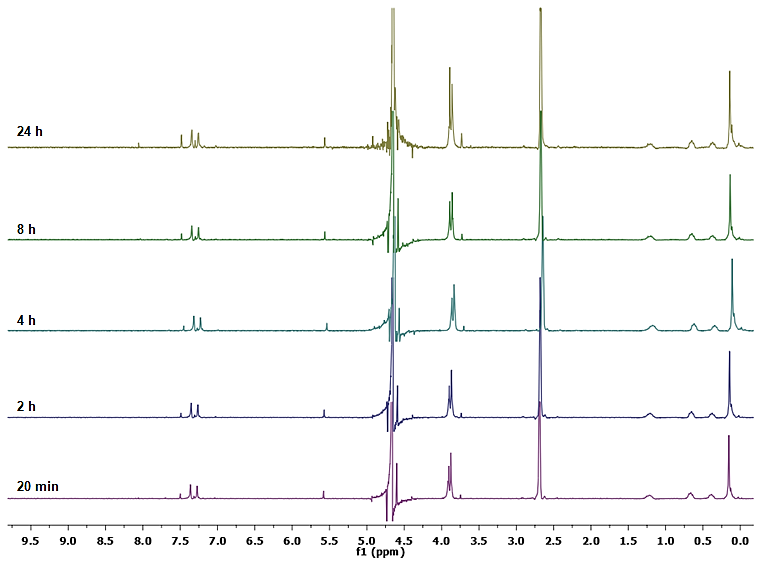


**Figure S7.** ^1^H-NMR study of G_1_Si(CH_2_MeImidAgCl)_4_ (1) in D_2_O:DMSO-d_6_ (1:1) at different times.


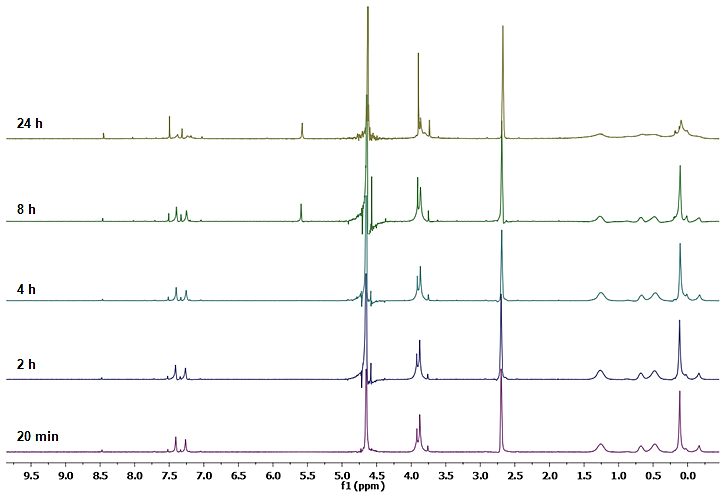


**Figure S8.** ^1^H-NMR study of G_2_Si(CH_2_MeImidAgCl)_8_ (2) in D_2_O:DMSO-d^6^ (1:1) at different times**.**


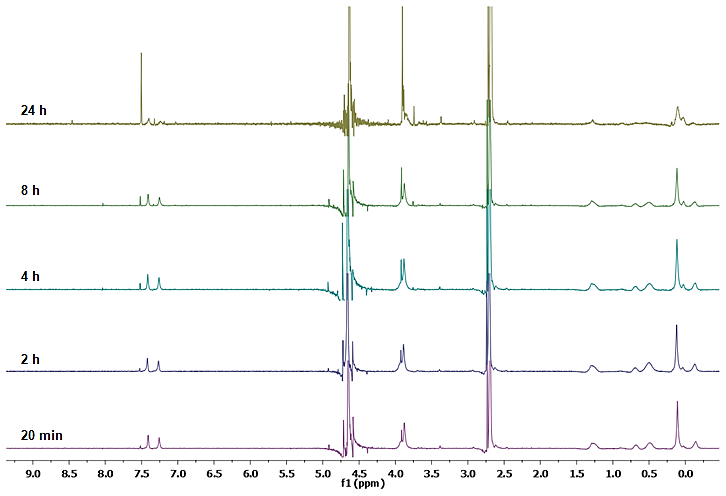


**Figure S9.** ^1^H-NMR study of G_3_Si(CH_2_MeImidAgCl)_16_ (3) in D_2_O:DMSO-d_6_ (1:1) at different times.

^
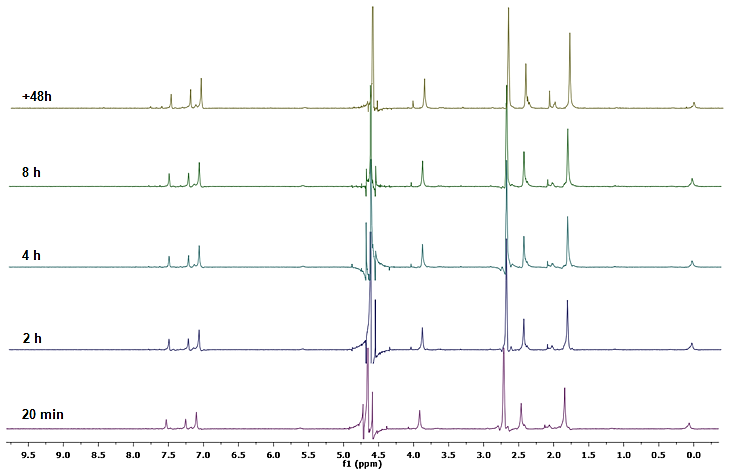
^

**Figure S10.** ^1^H-NMR study of G_1_Si(CH_2_MesImidAgCl)_4_ (4) in D_2_O:DMSO-d^6^ (1:1) at different times.


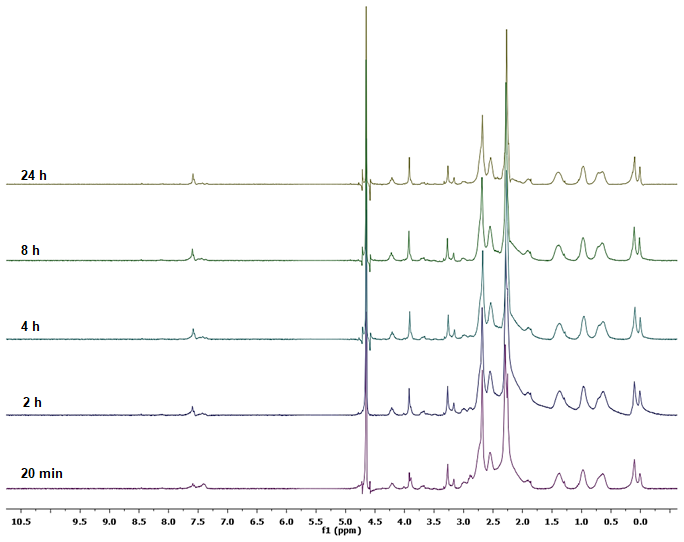


**Figure S11.** ^1^H-NMR study of AgClMeImidG_2_(S(CH_2_)_2_NMe_2_)_4_ (6) in D_2_O:DMSO-d^6^ (1:1) at different times**.**


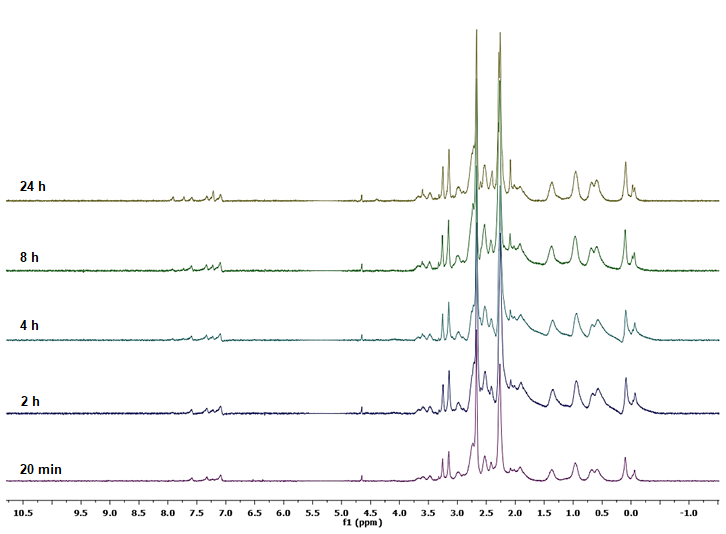


**Figure S12.** ^1^H-NMR study of AgClMesImidG_2_(S(CH_2_)_2_NMe_2_)_4_ (7) in D_2_O(WSAT):DMSO-d^6^ (1:1) at different times**.**

**11**

**15**

**Figure S13.** Schematic representations of (BrMeImid(CH_2_)_4_Si)_m_G_n_[OC_6_H_4_O]G_n_(Si(CH_2_)_4_ImidMeBr)_m_ Compounds 11-15.


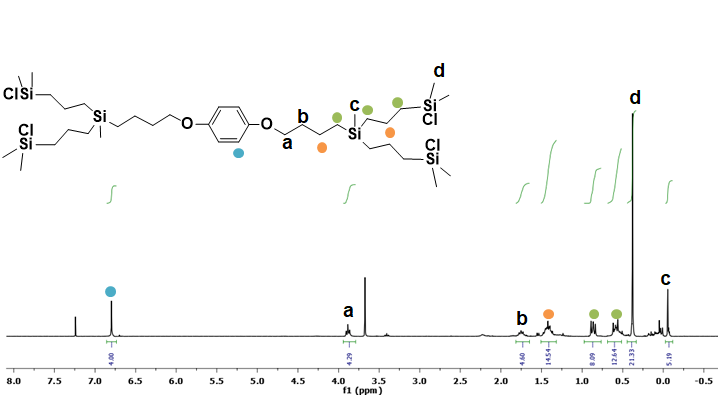


**Figure S14.** ^1^H-NMR of (Cl(CH_3_)_2_Si)_2_G_1_[OC_6_H_4_O]G_1_(Si(CH_3_)_2_Cl)_2_ (8) in CDCl_3_**_._**


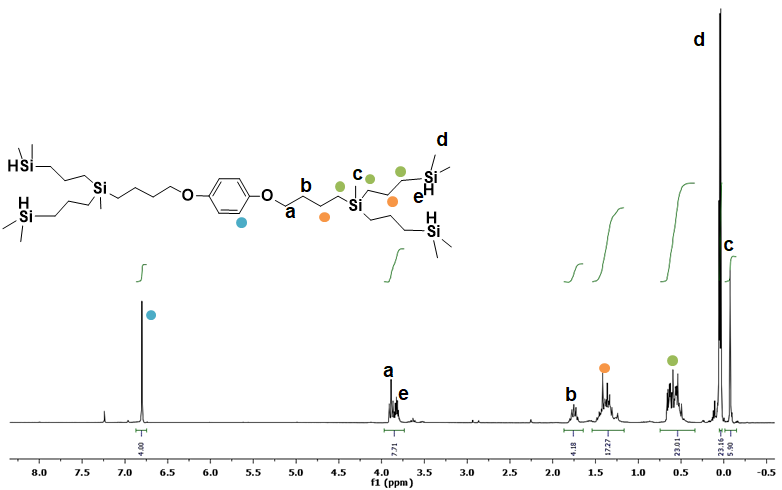


**Figure S15.** ^1^H-NMR of (H(CH_3_)_2_Si)_2_G_1_[OC_6_H_4_O]G_1_(Si(CH_3_)_2_H)_2_ (9) in CDCl_3_**_._**

_
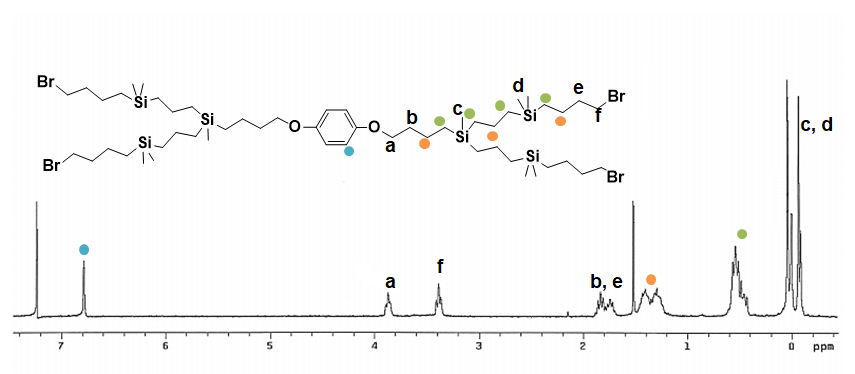
_

**Figure S16.** ^1^H-NMR of (Br(CH_2_)_4_Si)_2_G_1_[OC_6_H_4_O]G_1_(Si(CH_2_)_4_Br)_2_ (10) in CDCl_3_**_._**

_1
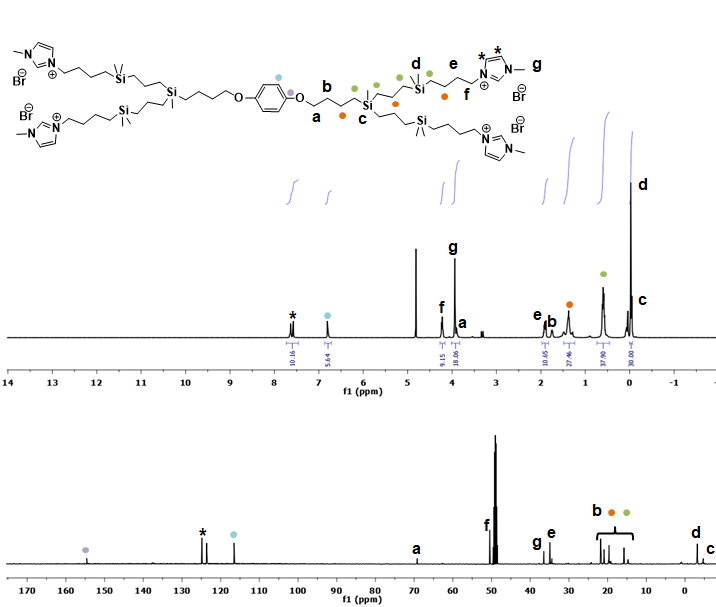
_

**Figure S17.** ^1^H-NMR and ^13^C{^1^H}-NMR of (BrMeImid(CH_2_)_4_Si)_2_G_1_[OC_6_H_4_O]G_1_(Si(CH_2_)_4_ImidMeBr)_2_ (11) in CD_3_OD**.**


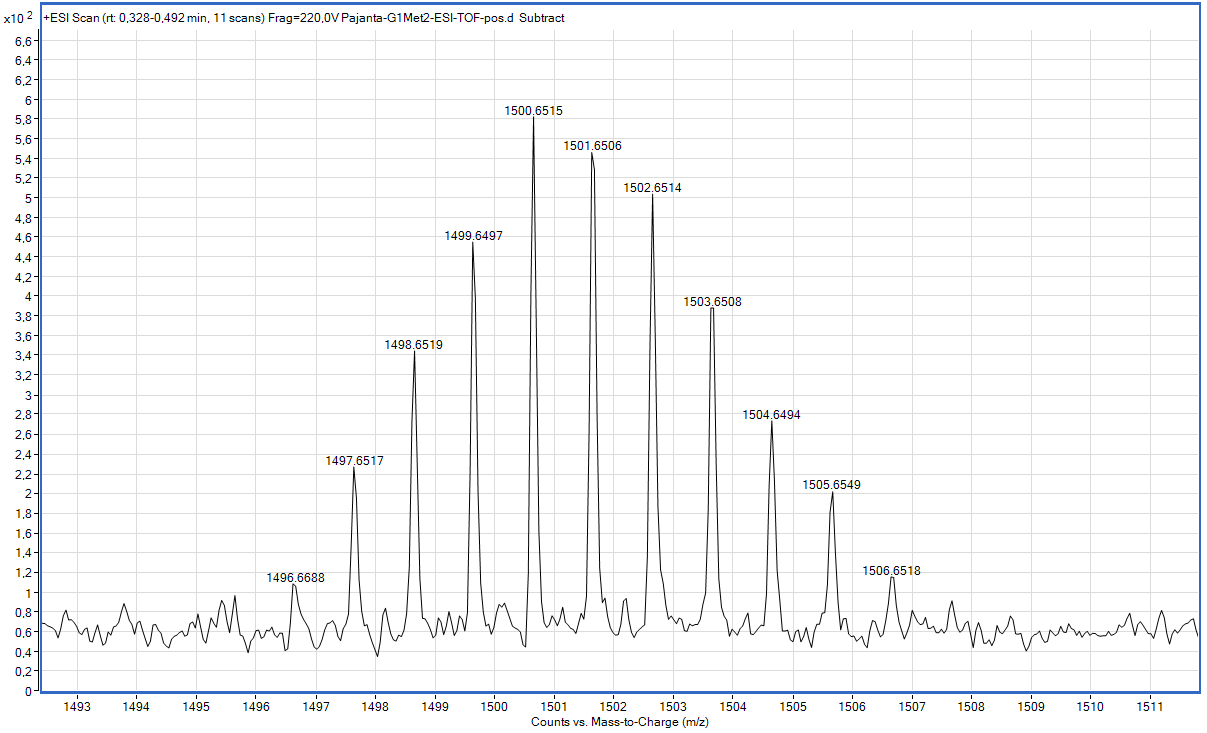


**[M-4Cl]^+4^**

**Figure S18.** ESI-TOF [M-Br]^+^ (BrMeImid(CH_2_)_4_Si)_2_G_1_[OC_6_H_4_O]G_1_(Si(CH_2_)_4_ImidMeBr)_2_ (11)**.**


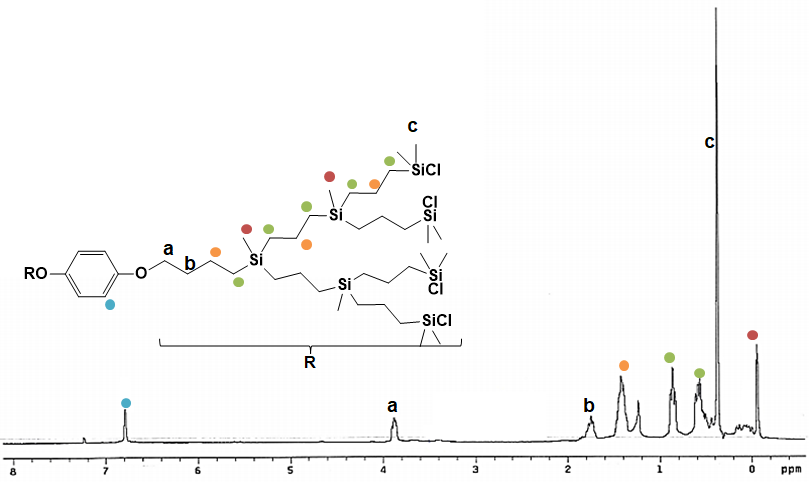


**Figure S19.** ^1^H-NMR of (Cl(CH_3_)_2_Si)_4_G_2_[OC_6_H_4_O]G_2_(Si(CH_3_)_2_Cl)_4_ (12) in CDCl_3_**_._**


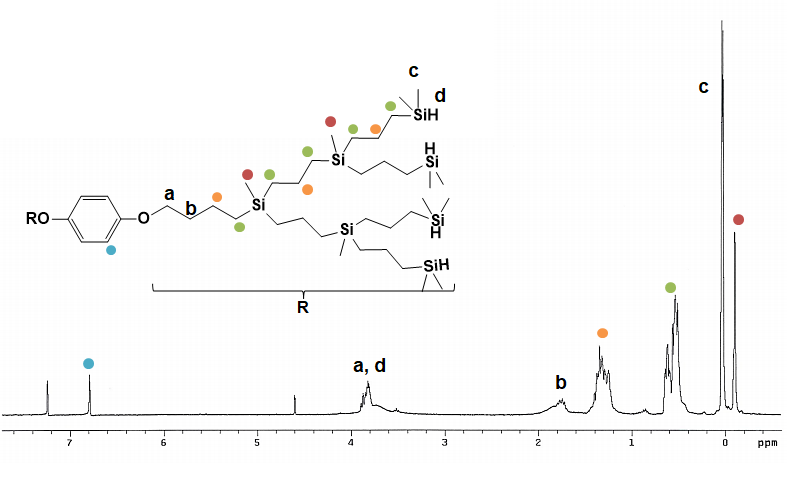


**Figure S20.** ^1^H-NMR of (H(CH_3_)_2_Si)_4_G_2_[OC_6_H_4_O]G_2_(Si(CH_3_)_2_H)_4_ (13) in CDCl_3_**_._**


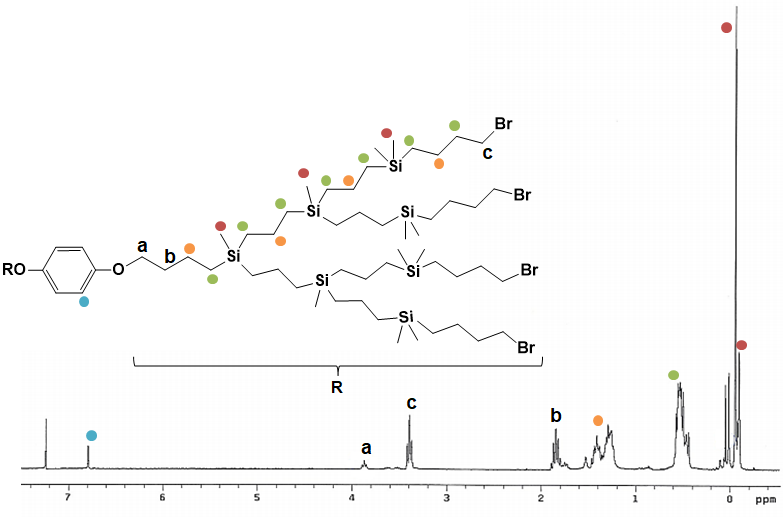


**Figure S21.** ^1^H-NMR of (Br(CH_2_)_4_Si)_4_G_2_[OC_6_H_4_O]G_2_(Si(CH_2_)_4_Br)_4_ (14) in CDCl_3_**_._**


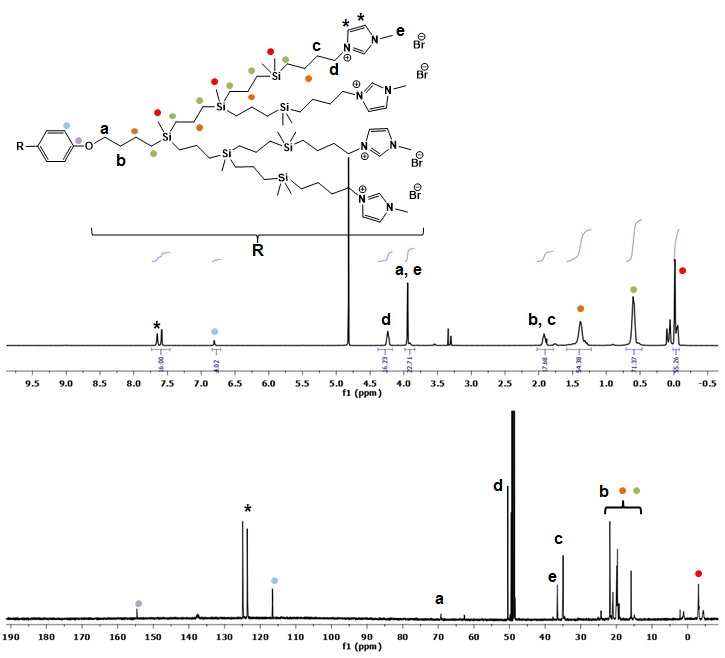

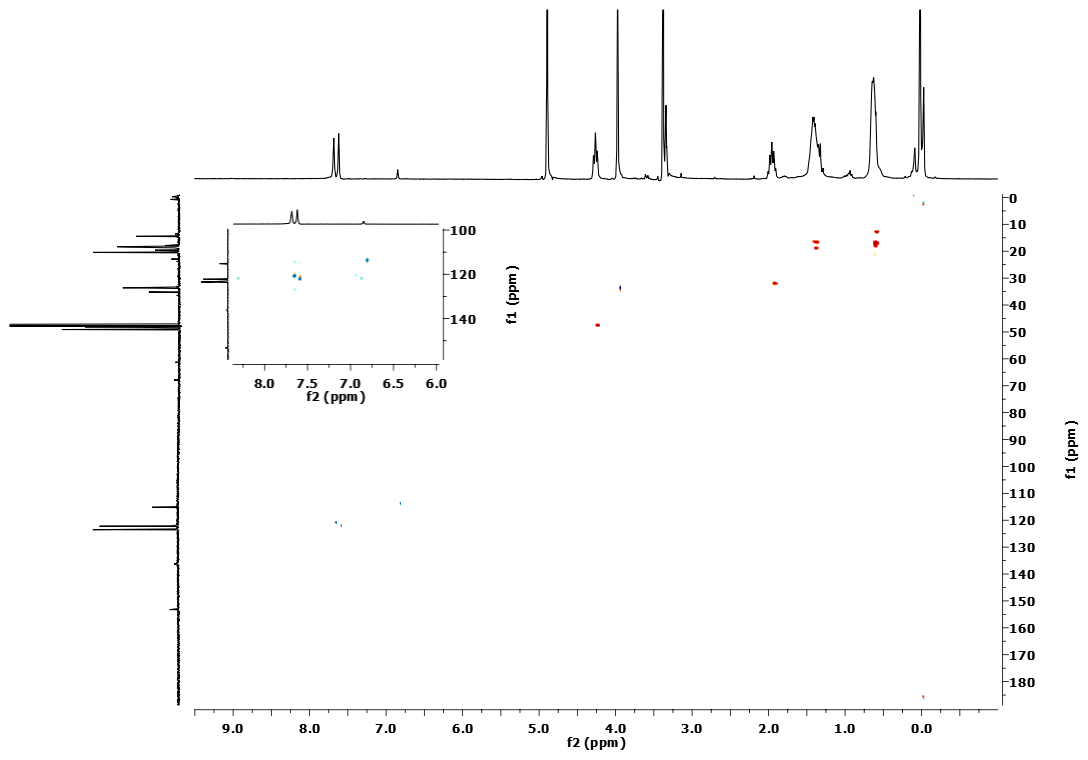


**Figure S22.** ^1^H-NMR, ^13^C{^1^H}-NMR and HSQC of (BrMeImidButSi)_4_G_2_[OC_6_H_4_O]G_2_(SiButImidMeBr)_4_ (15) in CD_3_OD**.**


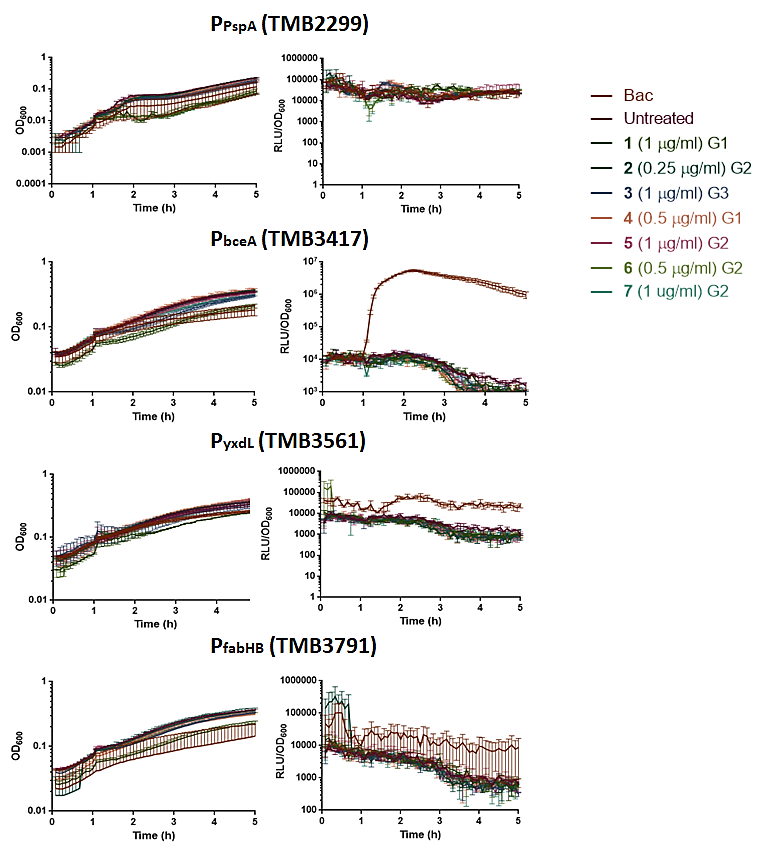


**Figure S23.** Biosensors induction in presence of dendrimers 1-5 and dendrons 6-7 containing Ag(I)- NHC carbenes in the periphery.


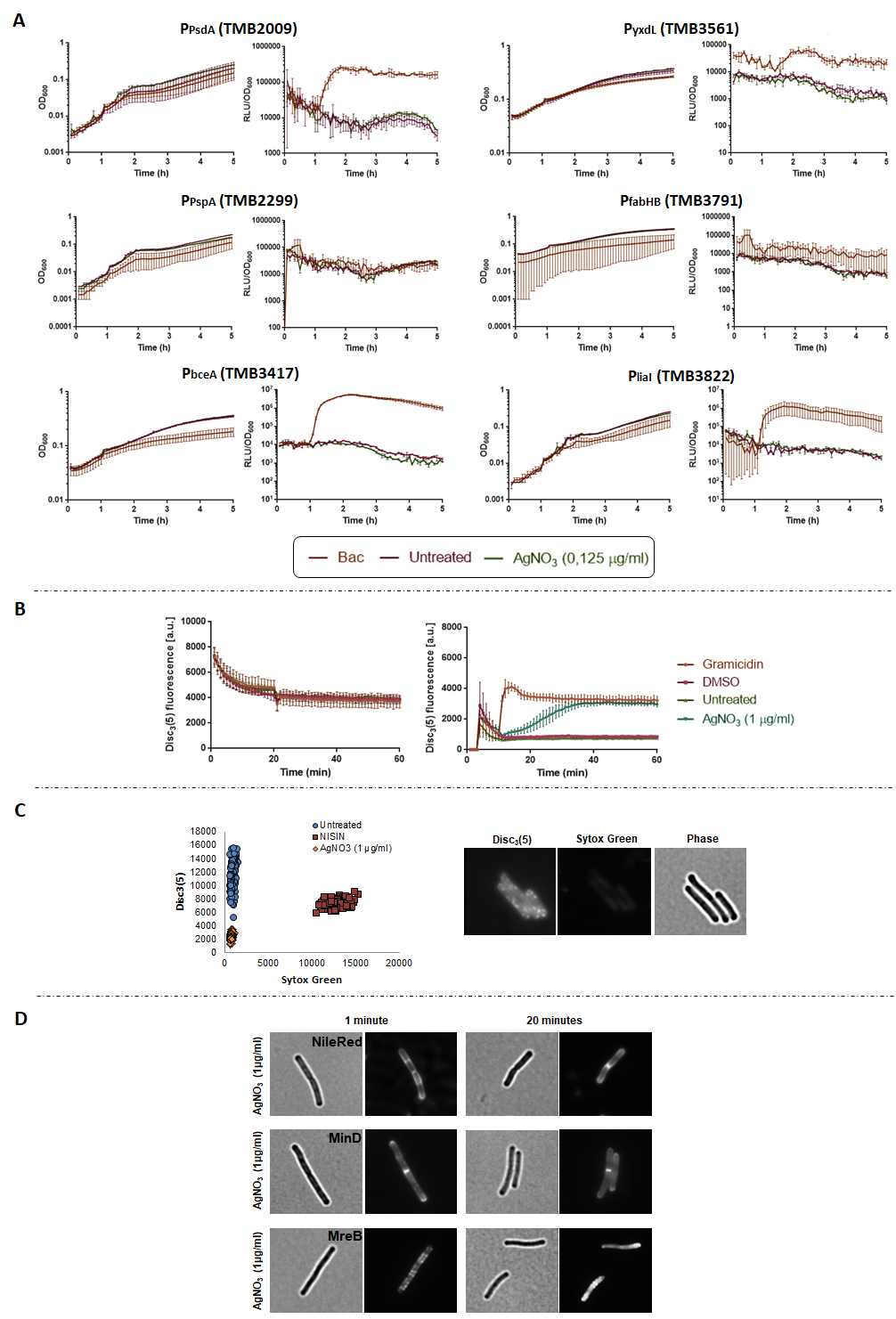


**Figure S24.** Results in presence of AgNO_3_: (**A**) Biosensor induction assay; (**B**) Fluorescence assay, control graph (left), depolarization graph (right); (**C**) Fluorescence assay. Microscopy; (**D**) NileRed and protein delocalization.


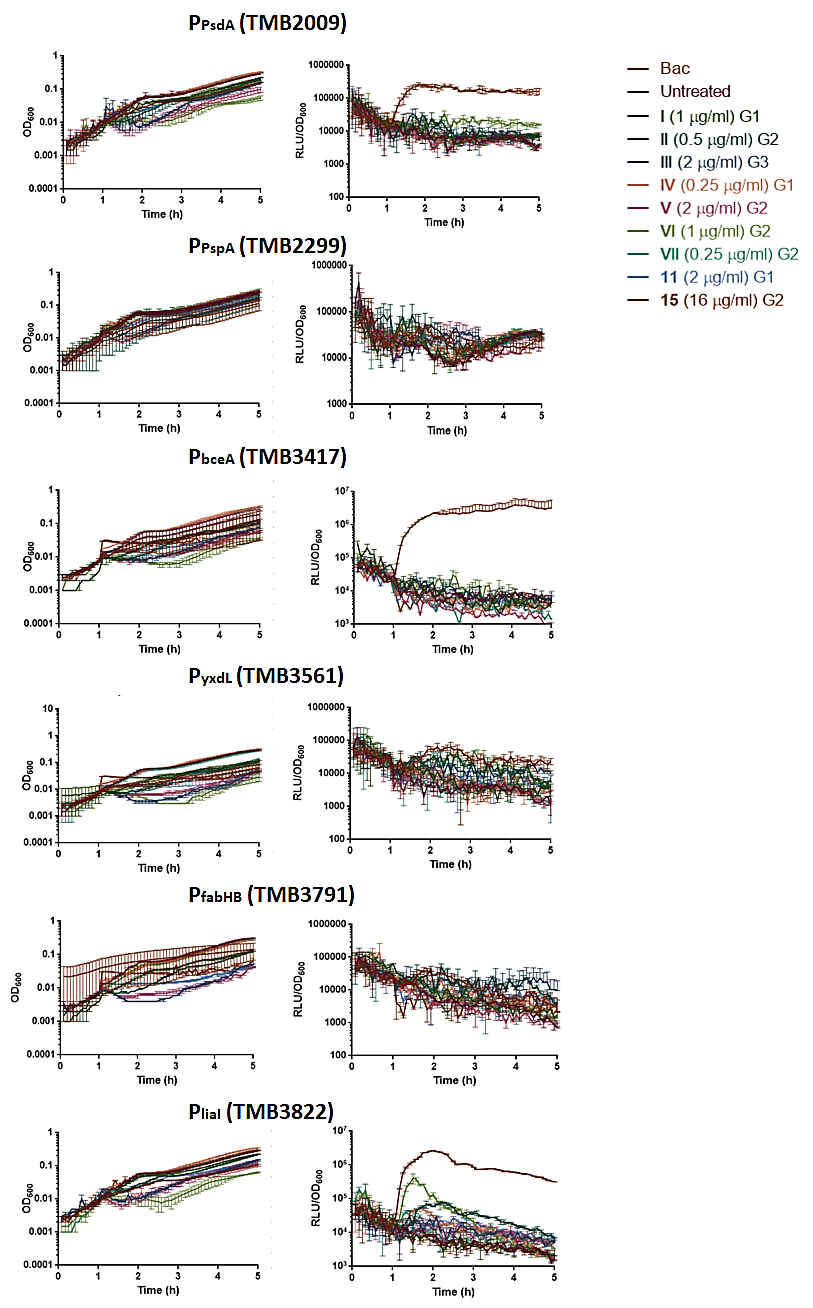


**Figure S25.** Biosensors induction in presence of dendrimers I-V, dendrons VI-VII and bow-ties 11, 15 containing imidazolium salts.


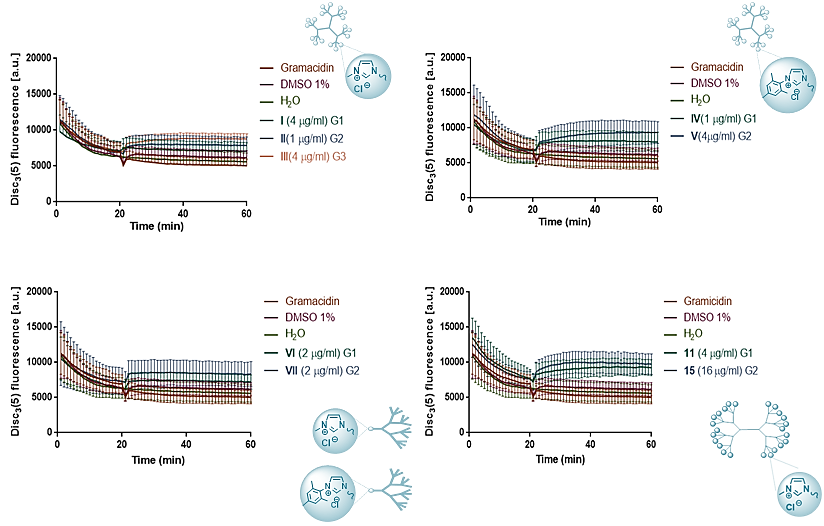


**Figure S26.** Disc_3_(5) fluorescence control graphs of cell membrane depolarization assay of *B. subtilis* WT168 in presence of compounds I-VII, 11 and 15.


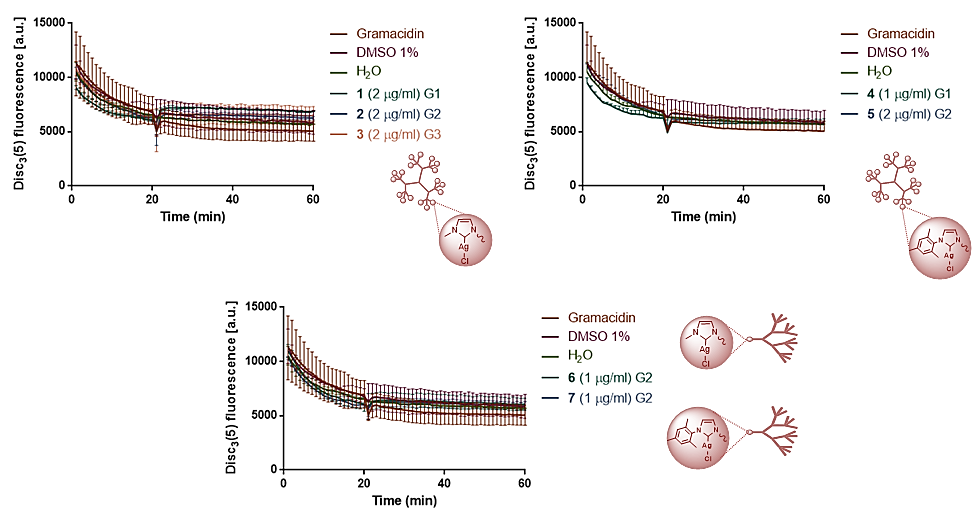


**Figure S27.** Disc_3_(5) fluorescence control graphs of cell membrane depolarization assay of *B. subtilis* WT168 in presence of compounds 1-7.


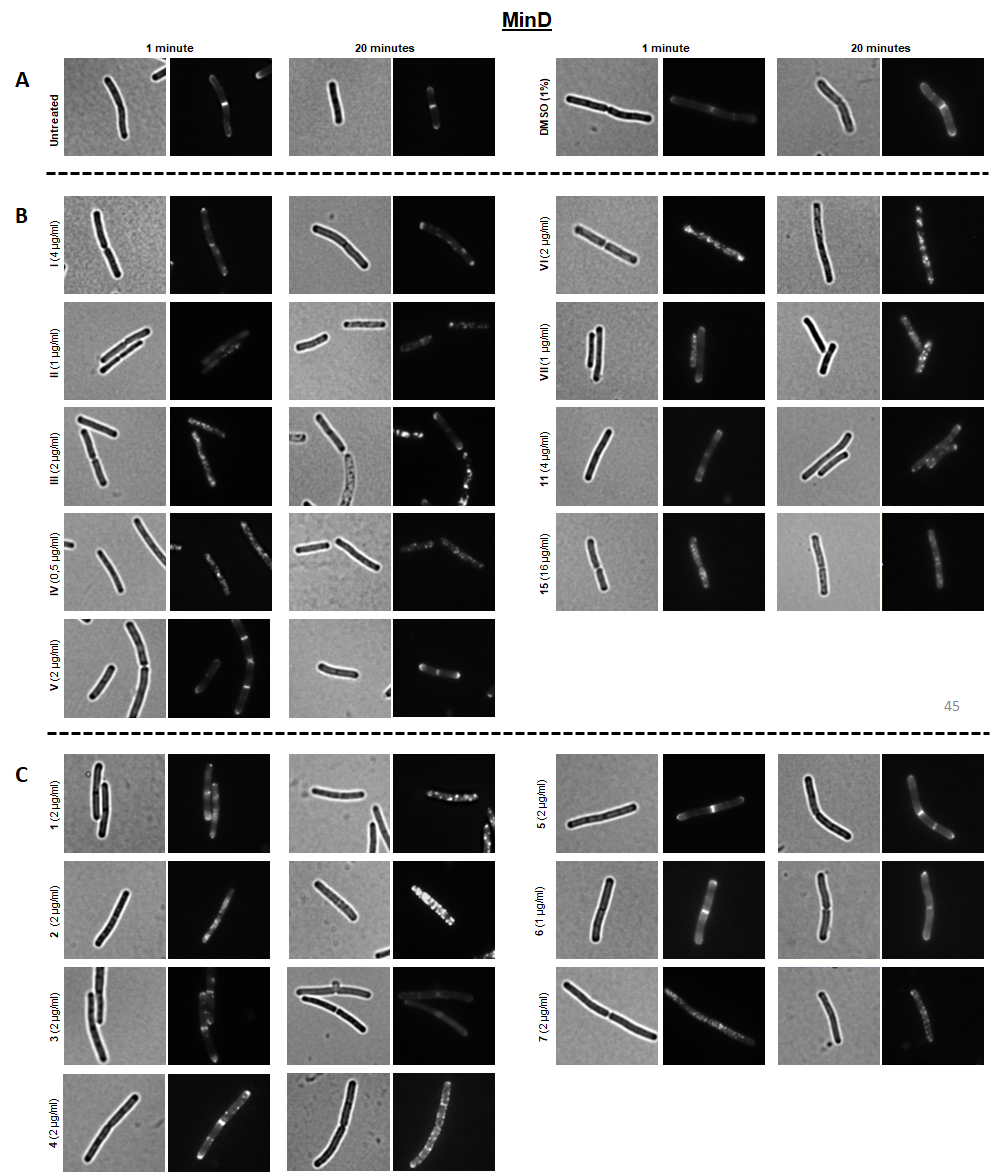


**Figure S28.** Representative microscopy images of MinD protein delocalization in presence of cationic (**B**) and Ag(I)-NHC (**C**) dendritic systems. Untreated and DMSO 1% are also depicted, to control the proper growth of bacteria (**A**).

**Table S1.** Comparative table detailing the activity of AgNO_3_ with the silver dendrimers 1-7 in molar ratio or *per* silver atom on *B. subtilis****.***

| **Compound** | ***MIC* (µg/ml)** | ***MIC* (µM)** | ***MIC* (µM) per number of Ag** |
| --- | --- | --- | --- |
| 1 | 1 | 0,72 | 2,88 |
| 2 | 1 | 0,32 | 2,59 |
| 3 | 2 | 0,31 | 4,93 |
| 4 | 1 | 0,55 | 2,22 |
| 5 | 4 | 1,02 | 8,16 |
| 6 | 1 | 0,98 | 0,98 |
| 7 | 1 | 0,89 | 0,89 |
| AgNO_3_ | 0,25 | 1,47 | 1,47 |
